# TRAPID: an efficient online tool for the functional and comparative analysis of *de novo* RNA-Seq transcriptomes

**DOI:** 10.1186/gb-2013-14-12-r134

**Published:** 2013-12-13

**Authors:** Michiel Van Bel, Sebastian Proost, Christophe Van Neste, Dieter Deforce, Yves Van de Peer, Klaas Vandepoele

**Affiliations:** 1 Department of Plant Systems Biology, VIB-Universiteit Gent, Technologiepark 927, B-9052 Gent, Belgium; 2 Department of Plant Biotechnology and Bioinformatics, Ghent University, B-9052 Gent, Belgium; 3 University of Potsdam, Institute of Biochemistry and Biology, Karl-Liebknecht-Straβe 24-25, Haus 20, 14476 Potsdam-Golm, Germany; 4 Max-Planck Institute of Molecular Plant Physiology, Am Mühlenberg 1, 14476 Potsdam-Golm, Germany; 5 Department of Pharmaceutics, Ghent University, B-9052 Gent, Belgium; 6 Department of Genetics, Genome Research Institute, University of Pretoria, Pretoria, South Africa

## Abstract

Transcriptome analysis through next-generation sequencing technologies allows the generation of detailed gene catalogs for non-model species, at the cost of new challenges with regards to computational requirements and bioinformatics expertise. Here, we present TRAPID, an online tool for the fast and efficient processing of assembled RNA-Seq transcriptome data, developed to mitigate these challenges. TRAPID offers high-throughput open reading frame detection, frameshift correction and includes a functional, comparative and phylogenetic toolbox, making use of 175 reference proteomes. Benchmarking and comparison against state-of-the-art transcript analysis tools reveals the efficiency and unique features of the TRAPID system. TRAPID is freely available at http://bioinformatics.psb.ugent.be/webtools/trapid/.

## Rationale

Technological advances in sequencing have made it possible to rapidly and cost-effectively take a snapshot of gene expression in a specific tissue or condition and have led to an explosion of transcriptome RNA-Seq data. With the Petabase barrier having been reached at the NCBI Short Read Archive (SRA) database at the end of 2012 [1], new approaches to deal with this surge in data quantity are required. For the plant kingdom alone, more than 4,200 transcriptome experiments covering more than 390 species are available at the SRA. Over 90% of these species do not have an available draft or complete genome sequence, making the data processing and biological interpretation a challenging task. In case a reference genome is available, the short reads can be processed using alignment-first (or align-then-assemble) methods that provide a genome-guided approach to study splice site junctions, identify new or alternative transcripts, or to quantify expression levels using known gene annotations [2]. In contrast, for species without a reference genome, assemble-then-align methods require that the millions of reads are first processed using *de novo* assembly before the reconstructed transcriptome is further characterized [3]. Examples of downstream analysis include the remapping of the input sequence reads from the different libraries to the assembled transcripts to quantify expression levels, the remapping of all reads to assess the genetic diversity within a genotype, or the alignment of the assembled transcripts against genome or transcripts sequences from closely-related species.

The development and improvement of *de novo* transcript assembly tools is an active research field and algorithms like OASES/Velvet, Trans-ABySS, and SOAPdenovo [3-6] provide efficient tools to reconstruct transcriptomes for non-model species starting from raw sequence reads. Despite the fact that both library normalization and increased sequencing depths (or higher coverage) will have a positive influence on the completeness of a transcriptome [7], most *de novo* transcriptome studies typically present gene catalogues where the number of transcripts after the assembly phase exceeds the estimated number of genes [8]. This pattern is mainly the result from redundancy caused by the presence of partial, unassembled, or highly heterozygous sequences. Despite these imperfections, *de novo* transcriptomes provide a sequence backbone for various non-model species and, in line with traditional genome projects, the detailed annotation of these transcript sequences is an essential step for downstream biological analysis.

Although the workflow to process transcriptome data is highly dependent on the type of analysis, functional annotation for the assembled transcripts is often generated using sequence similarity searches against a reference database. Clearly, the default application of large-scale sequence similarity searches against databases like NCBI or UniProtKB, which contain annotated proteins, drastically increases the amount of data that needs to be interpreted to derive functional annotations. Currently, systems like KEGG Automatic Annotation Server (KAAS) [9], Blast2GO [10], and T-ACE [11] provide tools for non-expert users to perform functional characterization of transcript sequences, but both the throughput as well as the quality of the reference datasets are important factors influencing the biological knowledge that can be extracted from non-model transcriptomes. Whereas systems like KAAS and Blast2GO can be operated through an Internet browser, T-ACE requires the installation of a PostgreSQL database on local hardware. Although both Blast2GO and T-ACE can derive functional annotations from a BLAST search against NCBI or through protein domain identification using InterProScan, the associated runtimes grow rapidly, hindering the efficient processing of a complete transcriptome dataset. Furthermore, the quality of the functional annotations of known sequences as well as the number of species or genes included in reference databases will have an impact on the success of translating transcript sequences into functional gene catalogs. Tools which apply the Gene Ontology (GO) controlled vocabulary benefit from the different functional levels embedded in the ontology structure, while systems like KEGG Orthology provide detailed information but only for a limited number of genes. Apart from functional annotations, the analysis of transcripts from non-model species using comparative genomics can also generate valuable information about conserved pathways, gene family expansions, species-specific genes, and genetic diversity [12-15]. However, performing such evolutionary analyses for thousands of transcripts is computationally expensive and user-friendly interfaces to compare *de novo* transcriptomes with high-quality reference genomes are still missing.

To address some of the issues inherent to the analysis of *de novo* transcriptomes, we present TRAPID, a web-based and high-throughput analysis pipeline that uses predefined reference databases. Available analyses include the automatic identification of coding sequences in transcripts, correcting frameshifts, assigning coding-sequences to multi-species gene families, performing transcript quality control, and generating functional annotations. Furthermore, detailed multiple sequence alignments and phylogenetic trees can easily be generated providing a comparative framework for the analysis of non-model transcriptomes. Finally, quantitative comparisons can be performed to study functional biases in transcriptome subsets derived from different tissues or conditions.

## General properties of the TRAPID transcriptome analysis tool

**Figure 1:**
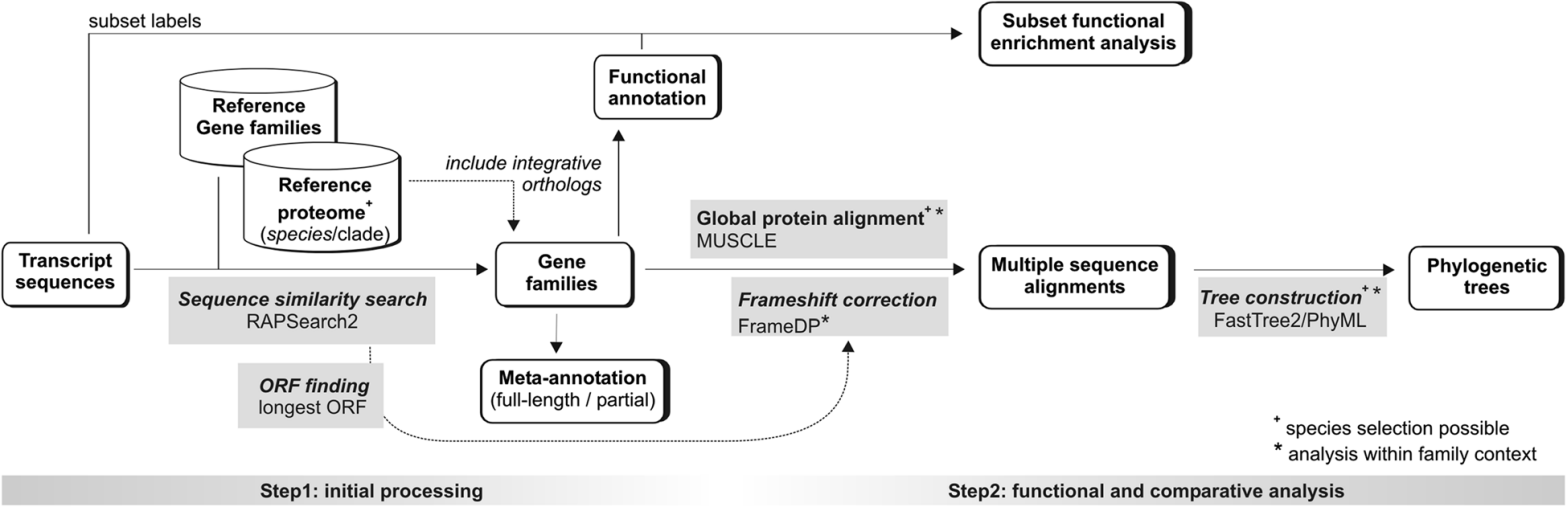
**Schematic overview of the TRAPID pipeline.** The TRAPID pipeline consists of two separate steps. The first one is a non-interactive processing step, during which all transcripts are assigned to gene families using a RAPSearch2 similarity search, followed by functional annotation transfer and meta-annotation assignment. The second step is interactive and directly commanded through the website interface. Here, the user has the ability to analyze his data using functional enrichment analyses, multiple sequence alignments, and phylogenetic trees.

To provide a web-based resource for the high-throughput processing of assembled transcriptomes derived from *de novo* RNA-Seq experiments or classical EST sequencing, a two-step procedure was developed. First, large-scale sequence similarity searches and open-reading frame (ORF) detection are combined to identify coding sequences, assign transcripts to gene families, identify partial/full length transcripts, and generate homology-based functional annotations. In a second step, detailed sequence analysis can be performed on-the-fly to correct frameshifts and study transcripts within an evolutionary context using multiple sequence alignments and phylogenetic trees (Figure 1). Although building a transcriptome analysis pipeline based on standard components for similarity searches and ORF detection is relatively straightforward, the large number of sequences and the fragmented nature of RNA-Seq data turns balancing the processing speed and quality into a highly challenging task. Furthermore, performing benchmarks to validate the designed computational protocol adds an additional layer of complexity. This section outlines the basic features of the TRAPID system while the following two sections focus on the implementation and benchmarking of specific analysis components. The last section provides a case study that illustrates how TRAPID can be used to quickly infer specific functional annotations for transcriptome subsets in a transparent and reproducible manner.

After the user creates a personal account, logs in into the TRAPID system and uploads a set of assembled transcripts (called an ‘experiment’), a sequence similarity search using RAPSearch2 [16] is executed against a specific protein database selected by the user (Figure 1). ‘Reference proteome’ databases refer to the full set of proteins for a given species or clade based on integrated genomes from PLAZA 2.5 [17] or OrthoMCL-DB version 5 [18]. As these genome resources also contain precomputed gene families, the TRAPID database ‘Gene family representatives’ contains one representative protein for each species present in a given gene family (see Methods). The reference proteomes makes it possible to select an appropriate specific taxonomic level and the full set of associated proteins based on the species the transcripts are originating from, while the latter provides a good alternative for the efficient processing of datasets within a broad taxonomic context, for example, in case no closely-related species with a reference proteome is available. Through the TRAPID website, we offer users a variety of reference protein databases and precomputed gene families (Table 1). Apart from 175 species-specific proteomes (25 from PLAZA and 150 from OrthoMCL-DB, including >2 million proteins) covering 25 plants, 115 non-plant eukaryotes, 36 Bacteria and 16 Archaea, 12 different clade, and two ‘Gene family representatives’ databases were generated to assign transcripts to families in a high-throughput manner. The provided reference proteomes and gene families offer a broad phylogenetic sample and a high-quality backbone for the comparative genomics features of TRAPID [19-21].

**Table 1:** Overview and content of the TRAPID reference databases

**Reference database**	**Functional annotation**	**Clade**	**#Species**	**#Proteins**	**Gene family information**
OrthoMCL-DB version 5	PFAM domains	All	150	1,398,546	OrthoMCL clustering
		Alveolata	15	98,796	
		Amoebozoa	4	41,930	
		Archaea	16	30,233	
		Bacteria	36	112,059	
		Euglenozoa	9	107,034	
		Eukaryota	98	1,256,264	
		Fungi	24	680,778	
		Metazoa	29	529,788	
PLAZA 2.5	Gene Ontology, InterPro domains	Viridiplantae (green plants)	25	780,667	TribeMCL clustering + integrative orthologs
		Angiosperms	18	671,950	
		Eudicots	13	480,106	
		Monocots	5	191,844	

The output of the sequence similarity searches is used to assign each transcript to a predefined gene family and to generate frame statistics to subsequently perform ORF detection. By default these frame statistics are submitted to a simple routine that extracts the associated longest ORF within the frame showing similarity with reference proteins (see Methods). However, this information is also used to predict whether specific transcripts contain putative frameshifts, which can, in a later stage and through the website, be automatically corrected using FrameDP, a self-training tool to predict peptide sequences in mature mRNA sequences [22]. The association of a transcript to a specific gene family is also used to facilitate the transfer of functional consensus Gene Ontology and protein domain information to transcripts. Finally, meta-information with regards to the length of the ORF of a transcript is generated, by comparing the ORF’s length to the average coding sequence length of the genes in the reference gene family.

**Figure 2:**
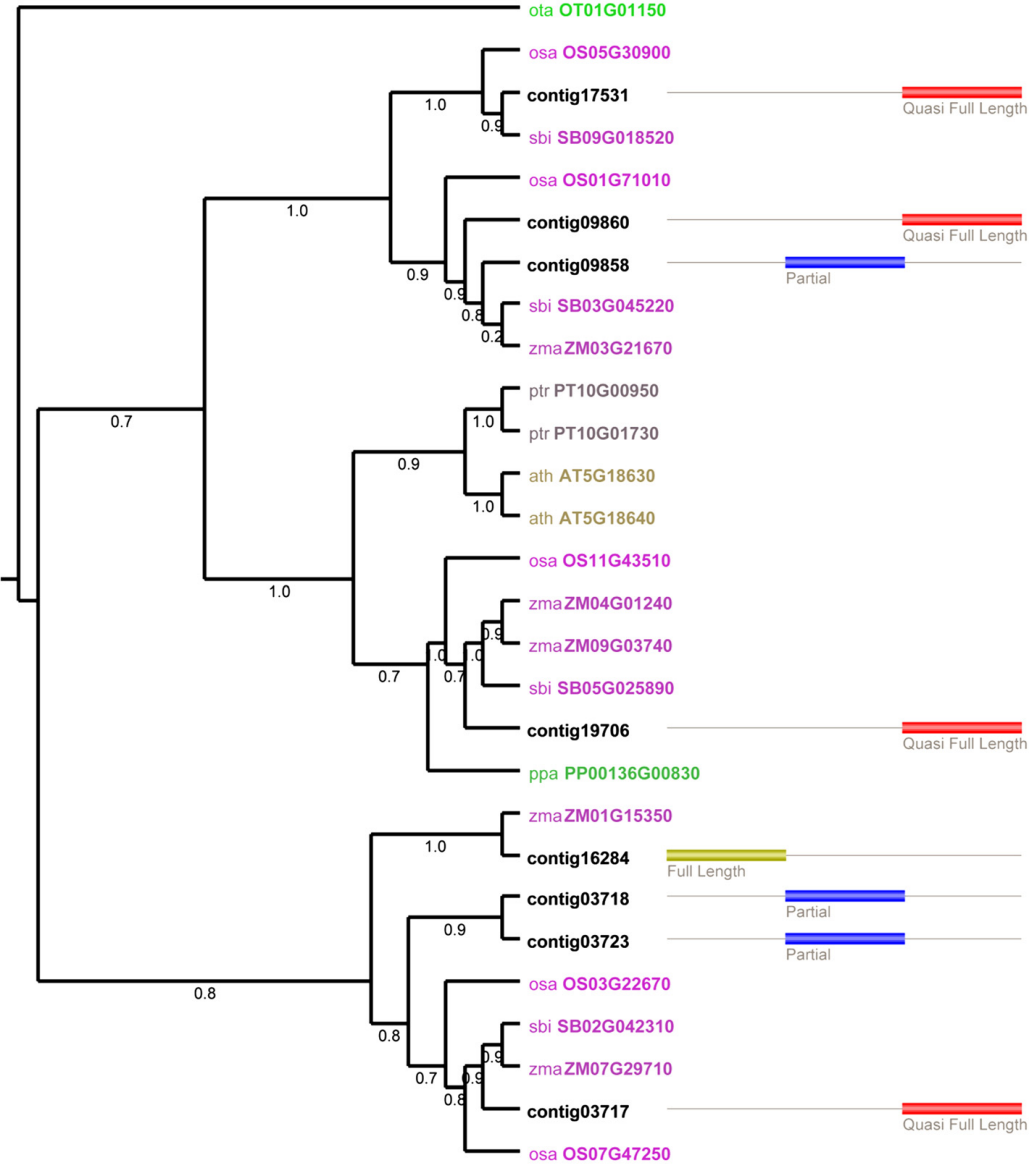
**Visualization of a phylogenetic tree with associated meta-annotation labels per transcript.** Cladogram based on a FastTree2 phylogenetic tree for the alpha/beta-Hydrolases superfamily (based on PLAZA 2.5 gene family HOM002165). Transcripts are marked in black, while colored gene identifiers refer to homologs from reference species: *Ostreococcus tauri* (ota), *Physcomitrella patens* (ppa), *Populus trichocarpa* (ptr), *Arabidopsis thaliana* (ath), *Oryza sativa* ssp. Japonica (osa), *Sorghum bicolor* (sbo), and *Zea mays* (zma). Meta-annotation for the different transcripts is visualized using the colored boxes on the right.

The second phase of the pipeline is performed interactively through the website, during which the transcripts associated with each gene family can be analyzed in more detail using homologous proteins from a set of reference species selected by the user. For transcripts that were flagged as potentially containing frameshifts, the user can execute FrameDP to putatively correct the transcript sequence and identify the correct ORF. Furthermore, based on the inferred coding sequences, per gene family a multiple sequence alignment is generated using MUSCLE [23] and the protein conservation of different transcripts with homologs from related species can be inspected using JalView [24]. Finally, after the application of an automatic editing routine for removing non-homologous alignment positions (see Methods), a phylogenetic tree is constructed using FastTree2 [25] or PhyML [26] providing a means to identify orthologous and paralogous gene relationships or trace putative allelic transcript variants (Figure 2).

Whereas the evolutionary analyses are based on predefined gene families from either OrthoMCL-DB or PLAZA, in some cases these families contain multiple out-paralogous sub-types (sub-clades within a family originating from an ancient gene duplication event predating most speciation events in the tree). As a consequence, some transcripts will be assigned to big gene families covering multiple genes, making phylogenetic analysis difficult. Therefore, in case a single species Reference Proteome is selected (Figure 1), it is possible to first assign transcripts to individual reference genes, for example, from a closely-related model species, and in a second phase build custom gene families through the inclusion of PLAZA integrative orthologs. These orthologs were identified using an ensemble method combining OrthoMCL, reconciled phylogenetic trees, colinearity information, and multispecies best hits and inparalogs (BHI) families [17], including inparalogs. In contrast to homologous gene families, families based on integrative orthology will contain a smaller number of genes, cover less outparalogs, and thus make downstream comparative analyses more feasible and the interpretation of complex families easier. Optionally, the user can also discard some species within a specific gene family in order to reduce the number of proteins before executing the phylogenetic tree construction routine. However, it is advisable to include as many species as possible in order to maintain a good taxon sampling and reduce phylogenetic error [27].

Apart from the functional annotation of individual transcripts, TRAPID also supports the quantitative analysis of experiment subsets using GO and protein domain enrichment statistics. Through the association of specific labels to sets of sequences, transcripts can be annotated with specific sample information (for example, tissue, developmental stage, control, or treatment condition) and be used to perform within-transcriptome functional analysis. Based on the integrated functional transcript annotation, enrichment analysis can subsequently be used to study the biological properties of specific experiment subsets or to compare the functional biases present in, for example, a treatment/control transcriptome experiment setup.

The TRAPID platform does not necessarily have to be the endpoint of a transcriptome analysis. In order to facilitate subsequent data analysis, multiple export functions have been added to the platform: all sequences, functional annotations, and phylogenetic data content can be downloaded by the user in a tab-delimited format. Collaborative work on a single transcriptome dataset is encouraged by the ability to share a TRAPID experiment between multiple users. The user who creates the experiment, is considered to be the ‘owner’ (and as such has the ability to empty or remove it), and this user can share his experiment with other TRAPID users (who can browse and edit). This prevents unnecessary data replication or sharing of user credentials.

## Evaluation of homology assignments

As shown in Figure 1, the first step is to assign each transcript to a predefined homologous gene family. Because transcriptome datasets for species lacking a reference genome sequence can contain more than 100,000 transcripts [8] (with a large fraction being fragments, allelic variants, splice variants, or highly expressed non-coding genes), the efficient processing of all these transcripts is essential to provide users with results within a reasonable timeframe. Two sequence similarity tools were considered: BLASTX [28] and RapSearch2 [16]. The transcript-to-family assignment results were compared using different protein reference databases with varying size, as the size of the database also influences the total runtime. BLASTX is often used to find proteins similar to a query gene in a large database, but requires a large amount of processing time. RapSearch2 was designed to perform the same searches but for short reads, and uses more efficient data structures to significantly speed up this process. Both tools were run using 1,000 randomly selected *Arabidopsis thaliana* transcripts against different databases containing all proteins from all species within a specific clade, and the correct assignment of a transcript to a family was evaluated together with the running time. In all evaluations the protein sequences of *Arabidopsis thaliana* and *Arabidopsis lyrata* were excluded from the database and the known assignments of *Arabidopsis thaliana* gene sequences to families from the PLAZA 2.5 database were used as a gold standard. Apart from reference databases containing all proteins for a specific species or clade, the ‘Gene family representatives’ database containing 32,294 proteins was also included in the test (see Methods). Assigning a transcript to a gene family was initially done with the 10 best similarity search hits using a simple majority-voting rule (Methods and Additional file 1). It is clear that both BLASTX and RapSearch2 assigned 87% to 98% of the transcripts to the correct gene family in all runs. For most reference databases the runtimes for RapSearch2 were approximately 10× lower compared to BLASTX, while overall, the gene family assignment quality was comparable. Increasing the reference database from one to multiple species (for example, from the Brassicales, which only contains *Carica papaya*, to Eudicots, covering 11 species) quickly increases the runtimes for both tools. However, better results with regards to the gene family assignment can be obtained by using a larger database. Various metrics, for example, taking only one or multiple hits into account, were evaluated to assign transcripts to families (Additional file 2). The best performance was generally achieved by considering the best hit when using species/clade reference databases and majority voting using the top five hits when using the ‘Gene family representatives’ database. To avoid overfitting (that is, a modeling error which occurs when a function or procedure is leading to a good fit with the sample data but a poor fit with new data) of this method to *Arabidopsis thaliana* transcripts, this benchmark was repeated using *Oryza sativa* spp. japonica (excluding *Oryza sativa* spp. japonica and *Oryza sativa* spp. indica from the databases) and *Vitis vinifera* (excluding *Vitis vinifera* from the databases), yielding similar results (Additional file 3). Although one would expect the correct assignment rate of a transcript to the corresponding gene family to decrease when the assembly quality of the input transcripts deteriorates, this is not always the case (Additional file 4). As such, even relatively short fragments of transcripts (for example, 50 to 100 nt) can be assigned to the correct gene family. Using manual inspection of the amino acid and sequence similarity information, the user is able to modify the association between a transcript and a family in case the automatic gene family assignment is deemed incorrect.

## Evaluation of ORF finding routine

In the absence of a reference genome, transcripts generated using *de novo* assembly of RNA-Seq reads frequently contain errors (for example, short insertions or deletions) and methods for the downstream analysis of coding sequences should be able to correct for potential frameshifts during ORF detection [29]. Although advanced self-learning algorithms (that is, methods that train themselves based on the input data provided by the user) such as FrameDP [22] exist to correct frameshifts during ORF prediction, running these tools on a complete RNA-Seq transcriptome on-the-fly is computationally unfeasible, even using multi-core or cluster hardware systems. Therefore, we implemented and evaluated a system to first perform the detection of putative frameshifts on all input sequences and subsequently only process these frameshift-containing sequences using FrameDP. This rationale is motivated by the observation that, when running FrameDP on complete plant transcriptomes, such as *Helianthus annuus* and *Pachysandra terminalis*[30,31], in only 3% to 15% of the input sequences a frameshift was identified that could be corrected.

Apart from gene family assignments, the Rapsearch2 output is also used to estimate if a frameshift is expected in an input transcript based on the output from the similarity search. For each input transcript the best hit in the reference database is selected and all alignments between this query and hit gene are evaluated. For each alignment the frame of the transcript hit is determined and if no frameshift is present, all alignments should report the same reading frame, which can immediately be used to extract the corresponding longest ORF (Figure 1). To evaluate this method to identify input transcripts containing frameshifts, we selected 1,000 transcripts from *Arabidopsis thaliana* containing no frameshifts and an equal amount of genes where one insert or deletion was artificially introduced at a random position in the coding sequence of the transcript (see Methods). Databases of various clades, each time excluding *Arabidopsis thaliana* and *Arabidopsis lyrata*, were used along with a database containing ‘Gene family representatives’ , to perform similarity searches. We found that, using these alignment-based frame statistics, 72.8% of all transcripts containing a frameshift were correctly identified, with only few (1.8%) false positives in the dataset lacking frameshifts (Additional file 5). To provide a good balance between global ORF quality and processing time, this method was integrated as the default procedure to identify frameshifts and subsequently correct them using FrameDP. The frame statistics suggest a fraction of frameshifts will be missed, especially when they occur near the 5′ or 3′ end of the gene due to relative small truncation of the full-length protein. As such the TRAPID system also provides an option for the user to run FrameDP on all transcripts within a family context.

## Comparison of TRAPID with Blast2GO and KAAS

A feature comparison between different publicly available web-tools for transcriptome analysis reveals that TRAPID has some unique properties (Table 2). The BLAST2GO [10] interface is commonly used to assign GO functional information to DNA or protein sequences by using either BLASTX or BLASTP, respectively. Although the BLAST2GO program can also be installed locally, reducing the run-time for the user at the cost of requiring in-house dedicated hardware, we compared TRAPID with the online Blast2GO interface in order to only compare web-based solutions. The KAAS platform [9] provides users with KEGG pathway information for a set of given sequences based on a BLAST bit scores. This functional information is complementary to other functional annotation systems such as GO or protein domains. In contrast to all other tools that focus solely on the functional annotation of input sequences, TRAPID provides additional functionalities to identify and correct frameshifts, perform ORF detection and downstream sequence and phylogenetic analyses (Table 2). The comparative genomics functionalities of TRAPID in particular offer the user an intuitive interface to inspect sequence conservation using multiple sequence alignments and to identify, using phylogenetic tree construction and an extensive set of reference genomes, orthologs in related species.

**Table 2:** Feature comparison web-based transcript analysis platforms

**Features**	**Blast2GO**^**a**^	**KAAS**	**TRAPID**
Sequence similarity search	NCBI BLAST	BLAST (bi-directional)	RAPSearch2
ORF finding	No	No	Yes
Frameshift correction	No	No	FrameDP
Reference database	NCBI non redundant database	Curated KEGG genes	OrthoMCL-DB version 5, PLAZA 2.5
Functional annotation	Gene Ontology, InterProScan, Enzyme codes, KEGG	KEGG (KEGG Orthology groups)	Gene Ontology, Protein domains (InterPro/PFAM)
Enrichment analysis	Yes	No	Yes
Protein alignments	No	No	MUSCLE
Phylogenetic trees	No	No	FastTree, PhyML
Others	advanced stand-alone graphical user interface	graphical pathway maps	ORF length meta-annotation, share experiments with other users

^a^Basic web-start version.

We conducted a series of benchmarks to assess both runtime and the transcript coverage of functional assignments for the different web-tools reported in Table 2. As the underlying hardware of the different tools and the load at the moment these experiments were executed are two parameters that cannot fully be corrected for, the results reported in Table 3 only give an approximation of the runtimes to process a complete transcriptome. As dataset we used 25,392 transcripts from *Panicum hallii*, a model for biofuel research [32]. For the TRAPID web-tool we used the Monocotyledon phylogenetic clade from the PLAZA 2.5 reference database (approximately 190,000 proteins), and for the other tools we used default parameters. The results in Table 3 highlight the much higher (up to 70×) required computational time of BLAST2GO compared to TRAPID or KAAS, while at the same time also being able to functionally annotate a higher amount of transcripts (20% for KAAS, 54% for TRAPID using GO, and 77% for BLAST2GO, respectively). Investigation of the GO terms which were uniquely assigned by BLAST2GO reveals that these annotations are less specific (Additional file 6A) and shows a large bias towards the Cellular Component GO category (Additional file 6B). Examples of very general annotations unique to BLAST2GO cover GO terms like ‘membrane’ , ‘intracellular’ , and ‘binding’. Furthermore, whereas TRAPID applies a conservative consensus model to transfer functions from families to transcript (see Methods), we observed that for many transcript receiving functional annotation solely through BLAST2GO, only one or a few of the BLAST hits support the inferred functions, representing a more liberal annotation approach.

**Table 3:** **Comparison of computation time and transcript coverage for different web-based transcript analysis platforms**^**a**^

**Size**	**KAAS**	**BLAST2GO**	**TRAPID (GO)**	**TRAPID (GF)**^**b**^
50	2 (16%)	29 (44%)	5 (50%)	5 (70%)
500	4 (20%)	232 (77%)	3 (54%)	3 (65%)
5000	11 (23%)	-	25 (54%)	25 (66%)
25392	32 (24%)	-	95 (54%)	95 (66%)

^a^Time is measured in minutes and numbers in parenthesis indicate the fraction of genes which received a GO functional annotation. The GO annotation for TRAPID was obtained through the protocol using the transfer from both the gene family and best-hit annotation. Missing values for BLAST2GO are due to runtimes larger than 24 h. The data is based on the 25,392 transcripts of *Panicum hallii*.

^b^GF indicates the fraction of transcripts assigned to a gene family.

## Detection of functional biases in transcriptome subsets using enrichment analysis

Apart from the general characterization of a complete transcriptome using various functional annotation systems, the detailed analysis of genes expressed in specific tissues or developmental stages can provide new insights about the underlying biological processes and their regulation. Again starting from the *Panicum hallii* transcriptome, we analyzed a set of transcripts showing distinct expression profiles in eight tissues for functional biases [32]. After processing all 25,392 contigs using the *Oryza sativa ssp. japonica* proteome as a reference and including integrative orthologs from the PLAZA 2.5 database, 16,748 (66%) transcripts were assigned to 9,860 gene families. Based on the results of expression clustering reported by Meyer and co-workers, 6,517 transcripts were tagged with a specific label (cluster 1 to 7) and GO enrichment analysis was performed for each subset. Whereas cluster 1, including transcripts with expression in stem-associated tissues, was significantly enriched for carbohydrate metabolism, cytoskeleton/cell wall organization, and shoot development (Additional file 7), seed-specific transcripts (cluster 5) included genes involved in the generation of precursor metabolites and energy, wax metabolism, and cuticle development (*P* value <0.05, hypergeometric distribution with Bonferroni correction). Transcripts showing differential expression in root and seedling (cluster 3) were enriched for translation, ribosome biogenesis, and rRNA metabolism, while leaf-specific expression (cluster 6) coincided with photosynthesis, energy metabolism, and multicellular organismal development, confirming previous results [32]. Finally, application of GO queries to tissue-specific subsets allows for the identification of transcriptional regulators involved in development. For example, searching for example ‘transcription factor activity’ on subset root (cluster 4) yields 21 transcription factors showing differential expression in root, including multiple CCAAT-binding, NAM, and bZIP proteins.

## Conclusion

TRAPID provides a publicly available tool to process *de novo* transcriptomes from animals, plants, fungi, and bacteria. This web-based application has been developed to offer a user-friendly interface to functionally characterize assembled transcript sequences and to initiate comparative genomics analyses, enabling scientists with a biological background to explore their non-model transcriptome data in an efficient and high-throughput manner.

## Methods

### Datasets, construction reference protein databases, and selection ‘gene family representatives’

The PLAZA 2.5 database was used as reference and as source for the Arabidopsis transcripts used in the benchmark experiments. The protein databases containing clade-specific content, for both the PLAZA 2.5 and OrthoMCL reference databases, were created by using NCBI Taxonomy [33] as reference. ‘Gene family representatives’ databases were constructed according to the procedure outlined by Van Bel et al. [17], in which for each species within a gene family a single gene is selected as representative. This is achieved by creating a graph with genes for nodes and BLAST bitscores for edges, and taking the most central and connected gene as representative. The *Pachysandra terminalis* dataset was retrieved from Vekemans et al. [30], *Helianthus annuus* and *Aquilegia formosa x Aquilegia pubescens* from TIGR Plant Transcript Assemblies [31]. *Panicum hallii* transcript sequences were retrieved from Meyer et al. [32] and contig sequences showing differential expression among tissues were isolated from Supporting Information, file S8.

### Similarity search, gene family assignment, and functional transfer using homology

We used RapSearch2 to search for protein hits for each query transcript (comparable to BLASTX), with a user-selectable e-value cutoff. In case the selected protein database consists of either species or clade specific proteins, then only the top protein hit is retained and the associated gene family for this protein is assigned to the transcript. In case the selected protein database consists of ‘Gene family representatives’ , then the top five protein hits are retained, and the gene family for the transcript is selected based on majority voting (see Additional file 2). The functional annotation for each transcript is transferred from its assigned gene family, the best similarity search hit, or a combination of both, depending on the choice of the user. In case the gene family is selected as basis for the functional annotation, the GO terms and protein domains are selected which constitute 50% or more of the size of the gene family. If not a single protein hit was detected during the similarity search, no gene family and no functional annotation is assigned to the transcript.

### Assigning frame information, detection and correction of potential frameshifts, and meta-annotation

From each alignment of the top protein hit the strand and frame is determined. If the same frame and strand is detected for each alignment then the longest Open Reading Frame (ORF) within this frame is stored. In case multiple alignments occurred with the target protein in different frames, the transcript was flagged as potentially containing a frameshift and the longest ORF in all possible frames was detected and retained. Using FrameDP version 1.0.3 [22] transcripts with expected frameshifts could be corrected. As a reference database all protein coding genes present in PLAZA 2.5 [17] or OrthoMCL-DB version 5 [18] were provided. FrameDP was configured to run with BLAST 2.2.17 (Expectation value: 1e-3, Open Gap Penalty: 9, Gap Extension Penalty: 2 and retaining only the 100 best hits) while the GC3 split training with three iterations was used. If the total number of selected transcripts is lower than 20, additional random transcripts are added in order to have a good background model. Other parameters were left at their default values. Test datasets that were generated to evaluate the frameshift correction procedure are available via the TRAPID FTP site [34].

The meta-annotation for all transcripts is determined by comparing the transcript length to the lengths of the coding sequences which constitute its associated gene family. In case no gene family was assigned to the transcript, or in case the associated gene family comprises less than five proteins, the transcript receives the label ‘No Information’ as meta-annotation. Otherwise, the lengths of the coding sequences from the gene family are ordered, and the longest 10% and shortest 10% are removed in order to reduce potential outliers within the reference data. Using the remaining lengths, the average and standard deviation are computed. If the transcript length is shorter than the average minus two standard deviations, the transcript receives the label ‘Partial’ as meta-annotation. If the transcript is longer, it receives the label ‘Quasi Full Length’ as meta-annotation. In case a transcript has meta annotation ‘Quasi Full Length’ , and its associated ORF has both a start and stop codon, than the meta annotation is changed to ‘Full Length’.

### Multiple sequence alignments and phylogenetic trees

Using MUSCLE the translated Coding Sequences (CDS) from transcripts belonging to the same gene family were aligned with amino acid sequences of homologous genes present in the reference database. MUSCLE provides a good balance between speed and accuracy [23] and in order to reduce the computation time for big gene families, the maximum number of iterations in the MUSCLE algorithm is fixed at three (all other settings are left at default). When building a phylogenetic tree, this multiple sequence alignment was edited following the same procedure as outlined in Proost et al. [21], where alignment columns containing gaps were removed when a gap was present in more than 10% of the sequences (stringent editing), as well as additional positions left and right from the gap. In case the stringent editing yields a stripped alignment with zero or only a few conserved alignment positions, the user can re-run the analysis using the relaxed editing option (gaps removed when present in at least 25% of the sequences). In addition, sequences flagged as ‘Partial’ can easily be discarded when performing phylogenetic tree construction. From this alignment phylogenetic trees can be generated using FastTree2 or PhyML, using the following parameters for protein sequences: ‘-wag and -gamma’ for FastTree, for PhyML WAG substitution model, empirical amino acid frequencies, default number of four relative substitution rate categories, maximum likelihood estimated gamma shape parameter. For both methods the number of bootstrap samples can be specified by the user. The Newick format of the trees was converted to PhyloXML [35] to allow the dynamic coloring of proteins within the phylogenetic tree.

## Implementation and availability

The website of the TRAPID platform was developed using CakePHP [36] with Flash and JavaScript used for visualizations, except for the Java programs used for the multiple sequence alignments and phylogenetic trees (JalView and Archaeopteryx, respectively). The backend of the online tool consists of a MySQL database [37] with custom Java programs and Perl scripts. A computing cluster (4 AMD Opteron(TM) Processor 6276 cores, 24 Gb of memory) is available allowing the simultaneous processing of up to four different datasets.

The TRAPID tool is available online [38]. The source code is also available online [39,40]. To install the software locally, users need to download and install third party software, as described in the TRAPID README file. General documentation (Additional file 8), a tutorial (Additional file 9) and an example dataset [32] are available on the TRAPID website.

## Abbreviations

GO: Gene Ontology; KAAS: KEGG Automatic Annotation Server; ORF: Open Reading Frame; SRA: Short Read Archive.

## Competing interests

The authors declare that they have no competing interests.

## Authors’ contributions

MVB developed and implemented the software (website and pipeline) and drafted large portions of the manuscript and the documentation. SP aided in the software development and pipeline design, performed the benchmark studies, and contributed to the tutorial and the manuscript. CVN tested the platform and contributed to the manuscript. DD and YVDP contributed to the manuscript. KV supervised the project and helped in designing of the pipeline, tested the platform, and contributed to the manuscript, tutorial and documentation. All authors read and approved the final manuscript.

## Supplementary Material

Additional file 1: Table S1 Benchmark homology assignments *A. thaliana.* Click here for file

Additional file 2: Table S2 Evaluation other metrics to assign transcripts to gene families. Click here for file

Additional file 3: Table S3 Benchmark homology assignments *O. sativa* and *V. vinifera.* Click here for file

Additional file 4: Table S4 Evaluation homology assignments using partial transcripts. Click here for file

Additional file 5: Table S5 Evaluation of frameshift detection. Click here for file

Additional file 6: Figure S1 Comparison of Gene Ontology functional annotations between BLAST2GO and TRAPID. Click here for file

Additional file 7: Figure S2 GO enrichment results for the plant *Panicum hallii* subset covering transcripts in stem-associated tissues. Click here for file

Additional file 8 TRAPID general documentation. Click here for file

Additional file 9 TRAPID tutorial. Click here for file
