# Supplementary material for: TRAPID: an efficient online tool for the functional and comparative analysis of de novo RNA-Seq transcriptomes
Source: Genome Biol. 2013 Dec 13;14(12):R134. doi: 10.1186/gb-2013-14-12-r134 (PMC4053847; doi:10.1186/gb-2013-14-12-r134)
Supplement: Additional file 1: Table S1 — Benchmark homology assignments A. thaliana. [file gb-2013-14-12-r134-S1.pdf]

Additional file 1. Supplementary Table 1. Evaluation BLASTX vs RAPSearch2

Input data	1)	1000 full-length *Arabidopsis thaliana* CDS sequences
Database	1)	Databases do not contain sequences from *Arabidopsis thaliana* and *Arabidopsis lyrata*
Evaluation	1)	determine whether, through the similarity search and taking X hits in consideration, the sequence is assigned to the correct (TribeMCL) gene family
Machine	1)	Evaluation was performed on the same machine, using only 1 core

BLASTX

#Top-hits	Brassicales	Malvids	Rosids	Eudicots	Angiosperm	VascularPla	LandPlants	GreenPlant	GF_REP
1	**948**	**974**	969	969	969	968	968	968	899
2	913	962	963	964	965	963	964	964	898
3	911	964	**975**	**975**	**975**	**975**	**975**	**975**	925
4	899	953	974	974	974	974	974	974	933
5	896	941	974	975	975	975	975	975	**943**
6	887	930	971	971	971	971	971	971	941
7	878	930	968	969	969	969	969	969	940
8	870	926	968	969	969	969	969	969	942
9	871	927	968	968	968	968	968	968	941
10	869	924	968	969	969	969	969	969	939
11	864	918	967	968	968	968	968	968	935
12	865	916	967	968	968	968	968	968	932
13	857	910	966	967	968	968	968	968	932
14	854	910	967	967	969	969	969	969	931
15	852	908	966	967	969	969	969	969	932
16	851	907	966	968	970	970	970	970	930
17	849	905	966	967	968	968	968	968	930

18	847	900	966	967	967	967	968	968	931
19	843	897	966	966	966	966	967	967	929
20	842	896	965	966	966	966	967	967	929
TIME	0h10m11s	0h24m29s	2h44m47s	2h56m33s	4h19m44s	4h30m33s	4h42m23s	5h11m55s	1h9m24s
TIME(s)	611	1469	9887	10593	15584	16233	16943	18715	4164

RAPSEARCH

#Top-hits	Brassicales	Malvids	Rosids	Eudicots	Angiosperm	VascularPla	LandPlants	GreenPlant	GF_REP
1	**931**	**965**	966	966	965	965	965	965	900
2	908	952	961	961	961	961	961	961	912
3	913	958	967	968	**968**	**968**	**968**	**968**	929
4	905	949	**968**	**968**	966	966	966	966	**936**
5	899	944	967	967	965	965	965	965	935
6	899	940	965	966	964	964	964	964	935
7	890	936	966	967	965	965	965	965	934
8	888	934	964	965	963	963	963	963	931
9	886	934	966	967	965	965	965	965	929
10	882	931	965	965	964	964	964	964	928
11	879	926	966	967	966	966	966	966	928
12	875	924	967	967	965	965	965	965	927
13	870	917	967	967	966	966	966	966	926
14	866	916	968	967	967	967	967	967	921
15	865	916	967	966	966	966	965	965	925
16	864	914	968	967	968	968	968	968	923
17	862	909	966	966	967	967	967	967	924
18	861	907	966	966	966	966	967	967	924
19	859	904	966	966	966	966	967	967	918
20	857	905	968	967	966	966	967	967	915
TIME	0h1m45s	0h3m8s	0h15m47s	0h17m18s	0h24m15s	0h25m1s	0h25m2s	0h25m38s	0h4m21s
TIME(s)	105	188	947	1038	1455	1501	1502	1538	261
SPEEDUP	5.8	7.8	10.4	10.2	10.7	10.8	11.3	12.2	16
