# Supplementary material for: TRAPID: an efficient online tool for the functional and comparative analysis of de novo RNA-Seq transcriptomes
Source: Genome Biol. 2013 Dec 13;14(12):R134. doi: 10.1186/gb-2013-14-12-r134 (PMC4053847; doi:10.1186/gb-2013-14-12-r134)
Supplement: Additional file 2: Table S2 — Evaluation other metrics to assign transcripts to gene families. [file gb-2013-14-12-r134-S2.pdf]

Additional file 2. Supplementary Table 2. Evaluation metrics

Input data	1)	1000 full-length *Arabidopsis thaliana* CDS sequences
Database	1)	Databases do not contain sequences from *Arabidopsis thaliana* and *Arabidopsis lyrata*
Evaluation	General	determine whether, through the similarity search and taking X hits in consideration, the sequence is assigned to the correct (TribeMCL) gene family
	1)	compare number of hits
	2)	compare bitscore sum of hits
	3)	compare normalized bitscore sum of hits
Machine	1)	Evaluation was performed on the same machine, using only 1 core

Hit count

#Top hits	Brassicales	Malvids	Rosids	Eudicots	Angiosperm	VascularPla	LandPlants	GreenPlant	GF_REP
1	**931**	**965**	966	966	965	965	965	965	900
2	908	952	961	961	961	961	961	961	912
3	913	958	967	968	**968**	**968**	**968**	**968**	929
4	905	949	**968**	**968**	966	966	966	966	**936**
5	899	944	967	967	965	965	965	965	935
6	899	940	965	966	964	964	964	964	935
7	890	936	966	967	965	965	965	965	934
8	888	934	964	965	963	963	963	963	931
9	886	934	966	967	965	965	965	965	929
10	882	931	965	965	964	964	964	964	928
11	879	926	966	967	966	966	966	966	928
12	875	924	967	967	965	965	965	965	927
13	870	917	967	967	966	966	966	966	926
14	866	916	968	967	967	967	967	967	921
15	865	916	967	966	966	966	965	965	925

16	864	914	968	967	968	968	968	968	923
17	862	909	966	966	967	967	967	967	924
18	861	907	966	966	966	966	967	967	924
19	859	904	966	966	966	966	967	967	918
20	857	905	968	967	966	966	967	967	915
	0h1m43s	0h3m44s	0h22m42s	0h24m41s	0h35m27s	0h37m8s	0h38m40s	0h43m31s	0h4m21s

Bitscore sum

#Top hits	Brassicales	Malvids	Rosids	Eudicots	Angiospern	VascularPla	LandPlants	GreenPlant	GF_REP
1	931	965	966	966	965	965	965	965	900
2	928	964	965	965	965	965	965	965	897
3	930	966	970	**971**	970	970	970	970	927
4	931	967	**971**	971	971	971	971	971	931
5	931	**968**	970	970	970	970	970	970	935
6	**934**	968	970	970	970	970	970	970	937
7	933	968	968	969	969	969	969	969	**942**
8	932	967	969	970	970	970	970	970	942
9	932	968	968	970	970	970	970	970	942
10	931	968	969	970	970	970	970	970	942
11	929	968	969	970	971	971	971	971	938
12	929	968	968	969	970	970	970	970	936
13	929	968	970	971	**972**	**972**	**972**	**972**	934
14	928	968	969	969	970	970	970	970	933
15	927	968	969	969	970	970	970	970	934
16	928	967	970	970	971	971	971	971	934
17	926	967	971	970	971	971	971	971	934
18	927	967	970	969	970	970	970	970	935
19	926	967	970	969	970	970	970	970	934
20	927	967	971	969	970	970	970	970	932
	0h1m43s	0h3m44s	0h22m42s	0h24m41s	0h35m27s	0h37m8s	0h38m40s	0h43m31s	0h4m21s

Bitscore sum normalized

#Top hits	Brassicales	Malvids	Rosids	Eudicots	Angiosperm	VascularPla	LandPlants	GreenPlant	GF_REP
1	**931**	**965**	**966**	**966**	**965**	**965**	**965**	**965**	**900**
2	928	964	965	965	965	965	965	965	897
3	927	962	964	964	963	963	963	963	888
4	924	962	961	961	960	960	960	960	877
5	923	963	961	960	960	960	960	960	871
6	920	963	957	958	958	958	958	958	861
7	921	961	953	956	956	956	956	956	851
8	919	958	951	950	950	950	950	950	846
9	919	955	944	946	947	947	947	947	840
10	918	951	939	943	944	944	944	944	829
11	918	949	935	938	938	938	938	938	827
12	918	950	928	929	932	932	932	932	814
13	919	946	919	923	928	928	928	928	811
14	919	947	909	912	920	920	920	920	807
15	918	945	897	898	910	910	909	909	802
16	918	942	892	893	905	904	904	904	791
17	918	941	886	888	902	902	900	900	782
18	918	941	886	884	897	895	896	896	775
19	917	939	887	880	891	889	889	889	772
20	918	938	881	878	884	882	883	881	771
	0h1m43s	0h3m44s	0h22m42s	0h24m41s	0h35m27s	0h37m8s	0h38m40s	0h43m31s	0h4m21s
