# Supplementary material for: TRAPID: an efficient online tool for the functional and comparative analysis of de novo RNA-Seq transcriptomes
Source: Genome Biol. 2013 Dec 13;14(12):R134. doi: 10.1186/gb-2013-14-12-r134 (PMC4053847; doi:10.1186/gb-2013-14-12-r134)
Supplement: Additional file 3: Table S3 — Benchmark homology assignments O. sativa and V. vinifera. [file gb-2013-14-12-r134-S3.pdf]

Additional file 3. Supplementary Table 3. Evaluation of *Oryza sativa ssp. Japonica* and *Vitis vinifera*

Input data	1000 *Oryza sativa ssp. Japonica* full length CDS sequences<br>1000 *Vitis vinifera* full length CDS sequences
Database	The databases do not contain *Oryza sativa ssp. Japonica* or *Oryza sativa ssp. Indica* sequences<br>The databases do not contain *Vitis vinifera* sequences
Evaluation	determine whether, through the similarity search and taking X hits in consideration,<br>the sequence is assigned to the correct (TribeMCL) gene family
Machine	Evaluation was performed on the same machine, using only 1 core

Oryza sativa ssp. Japonica

#Top hits	BEPClade	Monocots	Angiosperm	VascularPla	LandPlants	GreenPlant	GF_REP
1	895	942	936	936	936	936	863
2	875	935	928	928	928	928	848
3	873	934	931	931	931	931	878
4	867	932	930	930	930	930	881
5	862	929	929	929	929	929	875
6	856	924	929	929	929	929	871
7	854	917	925	925	925	925	863
8	847	915	923	923	923	923	859
9	849	912	924	924	924	924	859
10	848	911	923	923	923	923	857
11	845	910	923	923	923	923	852
12	842	909	922	922	922	922	851
13	839	908	921	921	921	921	851
14	835	907	921	921	921	921	848
15	835	906	918	918	918	918	850
16	834	904	917	917	917	917	848
17	832	902	916	916	916	916	845

18	830	897	916	916	916	916	844
19	829	898	915	915	915	915	844
20	826	897	915	915	915	915	843
	0h2m4s	0h6m37s	0h33m15s	0h34m50s	0h36m24s	0h40m56s	0h3m50s

Vitis vinifera

#Top hits	Eudicots	Angiosperm	VascularPla	LandPlants	GreenPlant	GF_REP
1	971	974	973	973	973	917
2	970	973	972	972	972	904
3	980	981	981	981	981	935
4	979	980	980	980	980	934
5	980	981	981	981	981	936
6	982	983	983	983	983	933
7	980	981	981	981	981	935
8	982	983	983	983	983	933
9	981	982	982	982	982	932
10	981	982	982	983	983	928
11	980	982	982	982	982	928
12	980	982	982	982	982	926
13	981	983	983	983	983	928
14	981	983	983	983	983	924
15	981	983	983	983	983	923
16	982	983	983	983	983	923
17	982	983	983	983	983	923
18	982	982	982	982	982	921
19	983	983	983	983	983	921
20	982	982	982	982	982	918
	0h19m55s	0h28m6s	0h29m18s	0h30m26s	0h33m49s	0h2m54s
