# Supplementary material for: TRAPID: an efficient online tool for the functional and comparative analysis of de novo RNA-Seq transcriptomes
Source: Genome Biol. 2013 Dec 13;14(12):R134. doi: 10.1186/gb-2013-14-12-r134 (PMC4053847; doi:10.1186/gb-2013-14-12-r134)
Supplement: Additional file 4: Table S4 — Evaluation homology assignments using partial transcripts. [file gb-2013-14-12-r134-S4.pdf]

Additional file 4. Supplementary Table 4. Evaluation of partial sequences

Input data	General	Partial sequences are further divided in 3 subcategories,<br>where each subcategory indicates whether 75%,50% or 25% of the original CDS length is retained<br>This is indicated by the PLXY% within the name of the dataset
	1)	1000 full-length *Arabidopsis thaliana* CDS sequences (FL100)
	2)	900 full-length *Arabidopsis thaliana* CDS sequences, 100 partial *A. thaliana* CDS sequences (FL90)
	3)	800 full-length *Arabidopsis thaliana* CDS sequences, 200 partial *A. thaliana* CDS sequences (FL80)
	4)	700 full-length *Arabidopsis thaliana* CDS sequences, 300 partial *A. thaliana* CDS sequences (FL70)
	5)	600 full-length *Arabidopsis thaliana* CDS sequences, 400 partial *A. thaliana* CDS sequences (FL60)
	6)	500 full-length *Arabidopsis thaliana* CDS sequences, 500 partial *A. thaliana* CDS sequences (FL50)
Database	1)	Databases do not contain sequences from *Arabidopsis thaliana* and *Arabidopsis lyrata*
Evaluation	1)	determine whether, through the similarity search and taking X hits in consideration,<br>the sequence is assigned to the correct (TribeMCL) gene family
Machine	1)	Evaluation was performed on the same machine, using only 1 core

Datset	Brassicales	Malvids	Rosids	Eudicots	Angiosperm	GreenPlants
FL100_PL75	922	920	898	898	900	899
FL100_PL50	922	920	898	898	900	899
FL100_PL25	922	920	898	898	900	899
FL90_PL75	921	921	899	898	901	900
FL90_PL50	919	923	902	902	904	903
FL90_PL25	920	920	900	900	902	902
FL80_PL75	919	918	894	893	898	897
FL80_PL50	927	925	906	906	907	909
FL80_PL25	932	928	904	903	906	908
FL70_PL75	923	922	902	902	903	902
FL70_PL50	921	926	906	905	907	907
FL70_PL25	921	922	903	903	903	906
FL60_PL75	922	928	901	900	903	903

FL60_PL50	926	930	911	911	913	913
FL60_PL25	933	933	909	910	911	914
FL50_PL75	922	922	903	903	907	904
FL50_PL50	926	931	912	911	915	914
FL50_PL25	932	929	913	913	916	919
