# Supplementary material for: TRAPID: an efficient online tool for the functional and comparative analysis of de novo RNA-Seq transcriptomes
Source: Genome Biol. 2013 Dec 13;14(12):R134. doi: 10.1186/gb-2013-14-12-r134 (PMC4053847; doi:10.1186/gb-2013-14-12-r134)
Supplement: Additional file 5: Table S5 — Evaluation of frameshift detection. [file gb-2013-14-12-r134-S5.pdf]

Additional file 5. Supplementary Table 5. Evaluation of frameshift predictions

Input data	Positive datase	1000 Arabidopsis thaliana genes with artificially introduced frameshifts in their ORF
	Negative datas	1000 unmodified Arabidopsis thaliana genes
Database	1)	Databases do not contain sequences from *Arabidopsis thaliana* and *Arabidopsis lyrata*
Evaluation	1)	determine how many frameshifts are positively identified, with as little false positives as possible
Machine	1)	Evaluation was performed on the same machine, using only 1 core

	**Eudicots**	**Green Plants**	**Gene family representatives**
Frameshifts found (positive)	726	726	652
Frameshifts found (negative)	17	18	15
FP-rate	1.70%	1.80%	1.50%
Recall	72.60%	72.60%	65.20%
Precision	97.71%	97.58%	97.75%
Accuracy	85.45%	85.40%	81.85%
