# Supplementary material for: TRAPID: an efficient online tool for the functional and comparative analysis of de novo RNA-Seq transcriptomes
Source: Genome Biol. 2013 Dec 13;14(12):R134. doi: 10.1186/gb-2013-14-12-r134 (PMC4053847; doi:10.1186/gb-2013-14-12-r134)

Additional file 6. Supplementary Figure 1. Comparison of Gene Ontology functional annotations between BLAST2GO and TRAPID.

The comparison of the GO annotations for 500 *Panicum hallii* transcripts. A distinction is made between GO annotations found exclusively by BLAST2GO (Blast2GO only), and those found by both TRAPID and BLAST2GO (shared). The three main GO categories, as well as a general overview are given. The X-axis contains the shortest-path-to-root (i.e. GO depth) for each GO term found. The Y-axis contains the number of transcripts that are annotated with a GO term from a certain depth.

A)

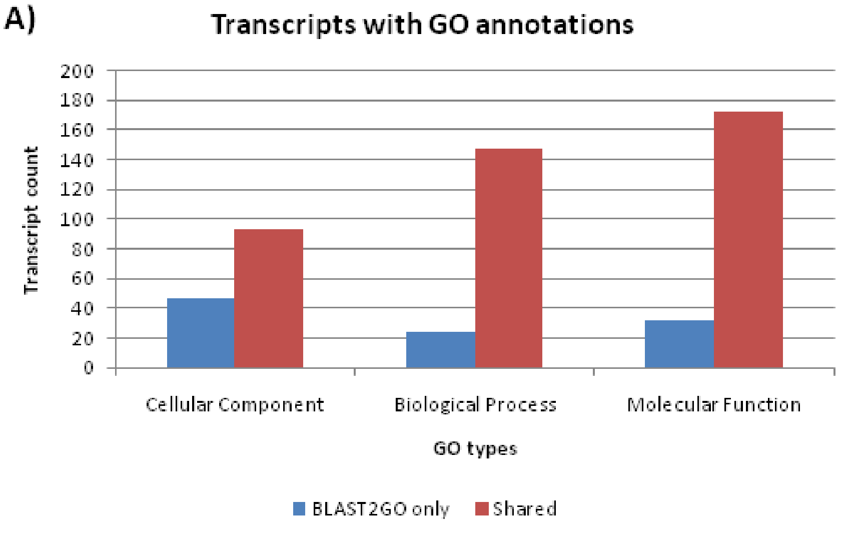

B)

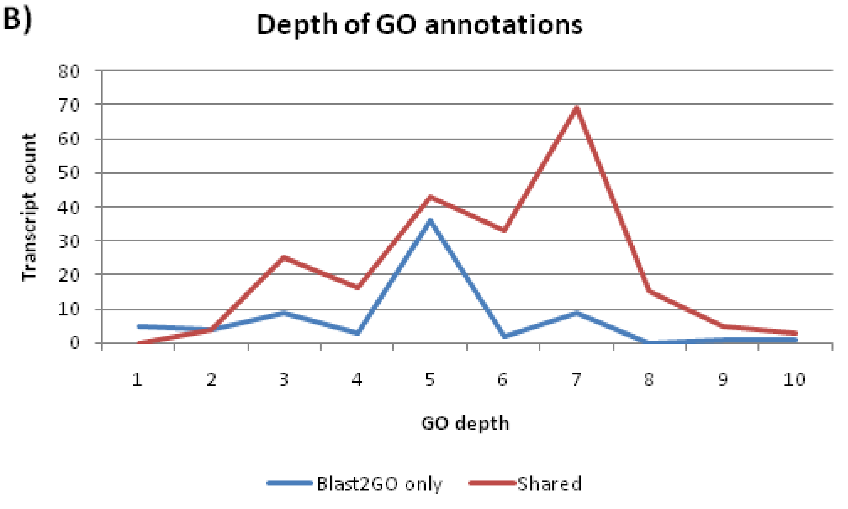

Supplement: Additional file 6: Figure S1 — Comparison of Gene Ontology functional annotations between BLAST2GO and TRAPID. [file gb-2013-14-12-r134-S6.pdf]
