# Supplementary material for: TRAPID: an efficient online tool for the functional and comparative analysis of de novo RNA-Seq transcriptomes
Source: Genome Biol. 2013 Dec 13;14(12):R134. doi: 10.1186/gb-2013-14-12-r134 (PMC4053847; doi:10.1186/gb-2013-14-12-r134)

Additional file 7. Supplementary Figure 2. GO enrichment results for the plant *Panicum hallii* subset covering transcripts in stem-associated tissues.

Biological Process enrichment plot for cluster 1 (stem-associated tissues) based on iORTHO *Oryza sativa* run. Whereas white (collapsed) boxes denote non-enriched functional GO terms, the intensity of the yellow shadings indicates the statistical significance of the functional overrepresentation. Clicking a specific functional GO term redirects the user to a page listing all associated transcripts.

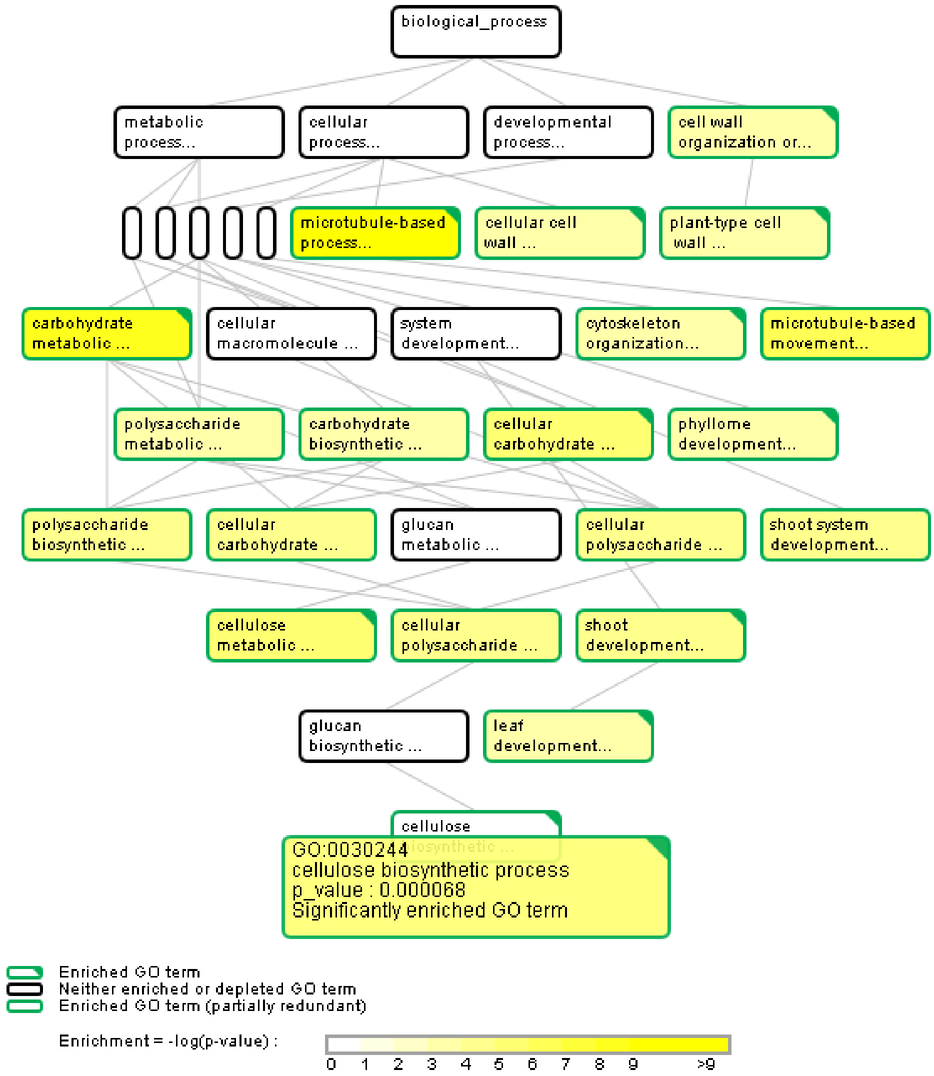

Supplement: Additional file 7: Figure S2 — GO enrichment results for the plant Panicum hallii subset covering transcripts in stem-associated tissues. [file gb-2013-14-12-r134-S7.pdf]
