# Supplementary material for: TRAPID: an efficient online tool for the functional and comparative analysis of de novo RNA-Seq transcriptomes
Source: Genome Biol. 2013 Dec 13;14(12):R134. doi: 10.1186/gb-2013-14-12-r134 (PMC4053847; doi:10.1186/gb-2013-14-12-r134)
Supplement: Additional file 8 — TRAPID general documentation. [file gb-2013-14-12-r134-S8.pdf]

TRAPID: Rapid Analysis of Transcriptome Data

Documentation

TRAPID General Documentation

Introduction & References

TRAPID is an online tool for the fast and efficient processing of assembled RNA-Seq transcriptome data. TRAPID offers high-throughput ORF detection, frameshift correction and includes a functional, comparative and phylogenetic toolbox, making use of 175 reference proteomes. The TRAPID platform Is available at **http://bioinformatics.psb.ugent.be/webtools/trapid**

Detailed information about the platform and the tools are provided in the different sections. In addition, we provide a detailed step-by-step Tutorial **http://bioinformatics.psb.ugent.be/webtools/trapid/documentation/tutorial**) to guide non-experts through the different steps of processing a complete transcriptome using TRAPID. Sample data including Panicum transcrips (from Meyer et al., 2012; see [1]) and subset labels can be found at **ftp://ftp.psb.ugent.be/pub/trapid/**.

Software pre-requisites:

- Supported browsers: Safari, Chrome, Firefox (most recent versions)
- JAVA installation (version JRE 6.0 or higher); this is required to successfully launch the JalView alignment editor and the Archeopteryx tree viewer.

[1] Meyer E, Logan TL, Juenger TE: Transcriptome analysis and gene expression atlas for Panicum hallii var. filipes, a diploid model for biofuel research. Plant J 2012, 70(5):879-890.

In case you publish results generated using TRAPID, please cite this paper:

TRAPID, an efficient online tool for the functional and comparative analysis of de novo RNA-Seq transcriptomes. Michiel Van Bel, Sebastian Proost, Christophe Van Neste, Dieter Deforce, Yves Van de Peer and Klaas Vandepoele. *journal*

In case you publish frameshift corrected sequences, MUSCLE multiple sequence alignments or phylogenetic trees generated using FastTree2 or PhyML, please also cite the corresponding papers:

- Gouzy J, Carrere S, Schiex T: **FrameDP: sensitive peptide detection on noisy matured sequences.** *Bioinformatics* 2009, 25:670-671.
- Edgar RC: **MUSCLE: multiple sequence alignment with high accuracy and high throughput.** *Nucleic Acids Res* 2004, 32:1792-1797.
- Guindon S, Dufayard JF, Lefort V, Anisimova M, Hordijk W, Gascuel O: **New algorithms and methods to estimate maximum-likelihood phylogenies: assessing the performance of PhyML 3.0.** *Syst Biol* 2010, 59:307-321.
- Price MN, Dehal PS, Arkin AP: **FastTree 2--approximately maximum-likelihood trees for large alignments.** *PLoS One* 2010, 5:e9490.

User Authentication

Data security is a necessary concern when dealing with online platforms and services. Through the use of user authentication no user has access to the data of any other user. User authentication is performed through username/password combination.

To acquire a username/password combination for the platform, select the Register option when visiting the TRAPID website. After supplying a valid email-address an associated password will be send to you. Using the email-address/password combination the user gains access to a 'to the user restricted' area within the TRAPID platform.

Step-by-step instructions on how to **create an account and log in** can be found in the tutorial.

Creating TRAPID Experiments

The transcriptome data should be uploaded to the TRAPID platform. Before doing this, it is important to note that, after authentication, the user has the ability to create different experiments for different transcriptome data sets, with a maximum of 5 experiments per user. So analyzing different transcriptome data sets at the same time is perfectly possible. The most important choice to be made here is what kind of reference database the user would like to use. The PLAZA reference database should be very good for transcriptome data sets from plant species, while the OrthoMCL reference database should be used for any other species, such as animals, fungi or bacteria.

Step-by-step instructions on how to **create an experiment** can be found in the tutorial.

Uploading transcript sequences and Job control

After the creation of a TRAPID experiment using the TRAPID website, the user should upload his transcriptome data to the platform. The transcriptome data should be made available as a multi-fasta file before upload to the server (max. size 30MB using the File upload option). In order to accommodate for the rather large file size associated with plain-text multi-fasta files, the uploaded file can also be compressed using zip or gzip. Fasta files, compressed or not, can also be uploaded by providing a URL to a specific transcript file (e.g. hosted at FTP site, public DropBox URL, etc); this option allows to upload bigger data sets (max. size 300MB). If the transcriptome data is split over several multi-fasta files, the user has the ability to continue uploading data (via traditional File upload or URLs) into his transcriptome data set before starting the processing phase. You will a receive an e-mail when your sequences have been successfully uploaded into the database.

During all TRAPID processing steps (upload, transcript processing, running frameshift correction or computing alignment/phylogenetic tree), users can check the Experiment Status to see if their job is queued, running or in error status. In case you want to cancel or stop your job, go to the Experiment Status page and modify the New status to Finished.

Step-by-step instructions on how to **upload data** can be found in the tutorial.

Perform transcript processing

The processing phase of the TRAPID platform is the next step, and necessary before any of the user custom analyses can be performed. This phase is initiated by selecting the Perform Initial Processing link on an experiment page. During this step, the user should consider the options carefully, as they may seriously impact the custom analyses.

- First and foremost is the choice of whether either a single species, a phylogenetic clade or the gene family representatives will be used for the similarity search (i.e. Database Type). A single species is a good choice if in the reference database a close relative of the transcriptome species is present. If a good encompassing phylogenetic clade is available, then this is also a solid choice. If none of the above, then the gene family representatives will provide a good sample distribution of the gene content within each reference database.
- Set an E-value cutoff for the RapSearch2 similarity search.
- Define the Gene Family Type: for PLAZA 2.5 this is Gene families (TribeMCL clusters) or Integrative Orthology (in case a single species was selected as Database Type), for OrthoMCL-DB this is Gene families (OrthoMCL clusters).
- Define how the functional annotation should be transferred from the family to the transcript level. In general, *transfer based on gene family* is the most conservative approach while *transfer based on best hit* is yielding a larger number of functionally annotated transcripts (47% and 51%, respectively, for the Panicum data set using clade = Monocots). Logically, combining both methods using *transfer from both GF and best hit* yields the largest fraction of annotated transcripts (54% Panicum datast).

After this step, the experiment will become available while the server performs the initial processing of the data. Again, you will a receive an e-mail when the processing is finished.

The processing of the data is sufficiently fast for normal transcriptome data sets. An test data set containing 90.000 transcripts can be processed in less than 3 hours, with approximately 28% of these transcripts assigned to gene families. For the Panicum data set (25392 transcripts) discussed in the manuscript, the complete processing (incl. upload as gzip file from public URL + transcript processing using clade = Monocots) takes around 1 hour (with 60% of transcripts assigned to gene families). The fraction of very short or very long transcripts will impact the total processing time during this initial phase.

Step-by-step instructions on how to **process transcripts** can be found in the tutorial.

Basic analyses

After the initial processing of the data has been performed, several new data types are available for the TRAPID experiment: gene families and functional annotation (GO categories or protein domains). Using these extra data types offers exciting new analyses to the user.

Step-by-step instructions on how to **browse these statistics** can be found in the tutorial.

General statistics

Offers a complete overview of ORF finding, gene family assignments, similarity search species information, meta-annotation and functional information.

Transcript information

#Transcripts	25392
Average sequence length	777.8
Average ORF length	538.2
#ORFs with a start codon	7157 (28.2%)
#ORFs with a stop codon	15677 (61.7%)

Frameshift information

#Transcripts with putative frameshift	2167 (8.5%)
#Transcripts with corrected frameshift	0 (0%)

Meta annotation information

#Meta annotation full-length	1810 (7.1%)
#Meta annotation quasi full-length	7379 (29.1%)
#Meta annotation partial	5922 (23.3%)
#Meta annotation no information	10281 (40.5%)

Similarity search information

Best similarity search hit for each transcript.

Sorghum bicolor	3771 (24.6%)
Zea mays	2684 (17.5%)
Oryza sativa ssp. japonica	1488 (9.7%)
Brachypodium distachyon	1478 (9.6%)
Oryza sativa ssp. indica	1392 (9.1%)
Theobroma cacao	457 (3%)
Vitis vinifera	448 (2.9%)
Fragaria vesca	338 (2.2%)
Populus trichocarpa	292 (1.9%)
Ricinus communis	276 (1.8%)

FIGURE 1 GENERAL STATISTICS EXAMPLE.

Subsets and labels

If the data set is comprised of transcriptome data from different sources (with sources indicating different tissues, developmental types or stress conditions), then the user has the ability to assign labels to the subsets. This is done through the *Import transcript labels* link on the experiment page and providing per label a list of transcript identifiers. Note that it is possible to assign multiple labels to one transcript. By assigning labels to transcripts, several new analyses become available, such as comparison of functional annotation between different subsets, or by computing functional enrichment.

Step-by-step instructions on how to **use subsets and labels** can be found in the tutorial.

Searching for data

The user has the ability to search for a number of possible data types within his selected experiment, through the search interface. Functional annotation can be searched for both through direct term identifiers (e.g. GO:0005509) or through the descriptions (e.g. Calcium ion binding).

Exporting data

The TRAPID platform allows the export of both the original data and the annotated and processed data of a user experiment. This data access is available under the *import/export data* header on an experiment page and includes structural ORF information, transcript/ORF/protein sequences and functional GO/InterPro information.

The export of the functional GO information has an extra column 'is_hidden', indicating whether a GO term is flagged as hidden, due to the presence of more informative GO codes in the GO graph for the given transcript.

The toolbox

On most pages (experiment/transcript/gene family/GO/protein domain) a toolbox is available which contains the most common analyses to be performed on the given

data object.

Frameshift correction

For transcripts that were flagged as potentially containing frameshifts, the user can execute FrameDP to putatively correct the transcript sequence and identify the correct ORF. FrameDP is a program which uses BLAST together with machine learning methods to build models which are used to test whether a sequence has a putative frameshift or not. The model is then used to correct the sequence (by inserting N-nucleotides at the necessary locations), which of course also directly has an impact on the associated Open Reading Frame (ORF). The drawback is however the exceptional long processing time. As such, the correction of frameshifts can only be done per gene family, and not on an entire transcript experimement.

The putative frameshifts are first detected during the "initial processing" phase, using a simple algorithm. The user has the ability to, on a gene family page, select these transcripts for FrameDP processing. If the total number of selected transcripts is lower than 20, additional random transcripts are added in order to have a good background model. Subsequently, all sequences are used for training and 'correction'.

Step-by-step instructions on how to **correct frameshifts using FrameDP** can be found in the tutorial.

Functional enrichment analysis

Apart from the functional annotation of individual transcripts, TRAPID also supports the quantitative analysis of experiment subsets using GO and protein domain enrichment statistics. Through the association of specific labels to (sub-)sets of sequences, transcripts can be annotated with specific sample information (e.g. tissue, developmental stage, control or treatment condition) and be used to perform within-transcriptome functional analysis.

Specific comparative analyses than can be performed using subsets are:

- GO enrichment (subset versus all; hypergeometric distribution): bar chart, table and enrichment GO graph output

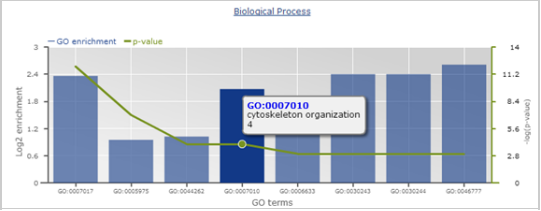

FIGURE 2 GO ENRICHMENT.

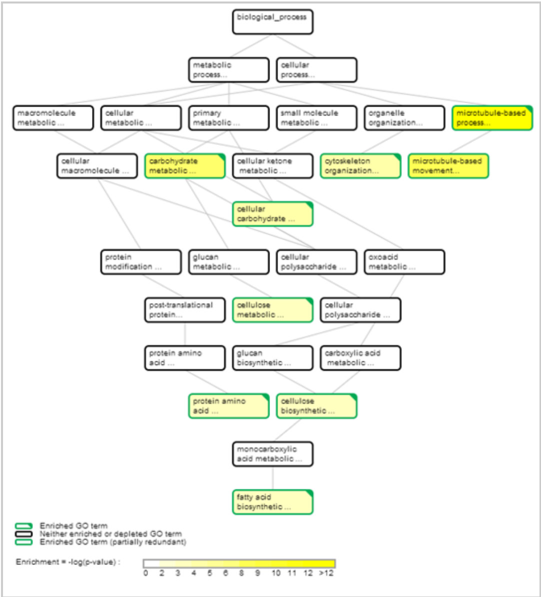

FIGURE 3 GO GRAPH.

- GO ratios (table) calculates GO frequencies between subsets. Also includes subset-specific GO annotations
- GO ratios (table) calculates GO frequencies between subsets. Also includes subset-specific GO annotations
- Protein domain enrichment (subset

versus all; hypergeometric distribution)
- Protein domain ratios between subsets
- Different subsets - Venn diagrams

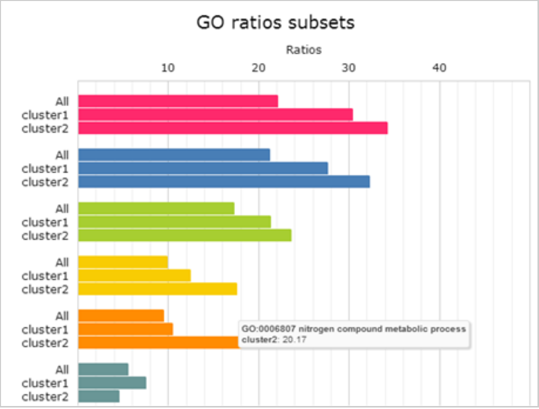

FIGURE 4 GO RATIOS.

Multiple sequence alignment

Starting from a selected transcript, the user has the ability to create an amino acid multiple sequence alignment (MSA) within a gene family context. As such, the user can create an MSA containing the transcripts within a gene family together with a selection of coding sequences from the reference database. This tool is accessible from the toolbox from a gene family. The MSA is created using MUSCLE

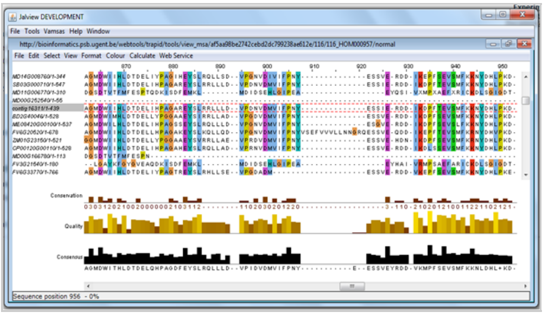

FIGURE 5 EXAMPLE MULTIPLE SEQUENCE ALIGNMENT. Panicum data set, transcript contig16311 in family HOM000957 covering 117 genes from 25 species.

(**http://www.ebi.ac.uk/Tools/msa/muscle/**), a tool which delivers a good balance between speed and accuracy (Edgar 2004 Nucleic Acids Res. 2004; 32(5): 1792-1797). In order to reduce the computation time, the maximum number of iterations in the MUSCLE algorithm is fixed at three. All other settings are left at default.

After the MSA has been created, the user has the ability to view this alignment using JalView, or to download the MSA and investigate it using a different tool.

Phylogenetic trees

The user has, similar to the multiple sequence alignment, also the ability to create a phylogenetic tree within a gene family context (see previous section). In order to create a phylogenetic tree, the system needs this MSA for the tree building algorithm. This step, however, is done automatically by the TRAPID system in case no previous MSA is present for the indicated gene family.

In order to create phylogenetic trees which are less dependent on putative large gaps, the standard MSA is transformed to a *stripped* MSA. In this stripped MSA the alignment length is reduced by removing all positions (for every gene/transcript sequence) for which a certain fraction (0.10 for stringent editing, 0.25 for relaxed editing) is a gap. As such, all gaps introduced by a small number of sequences will be removed.

In case the stringent editing yields a stripped MSA with zero or only a few conserved alignment positions, please re-run the tree analysis using the relaxed editing option, which will yield

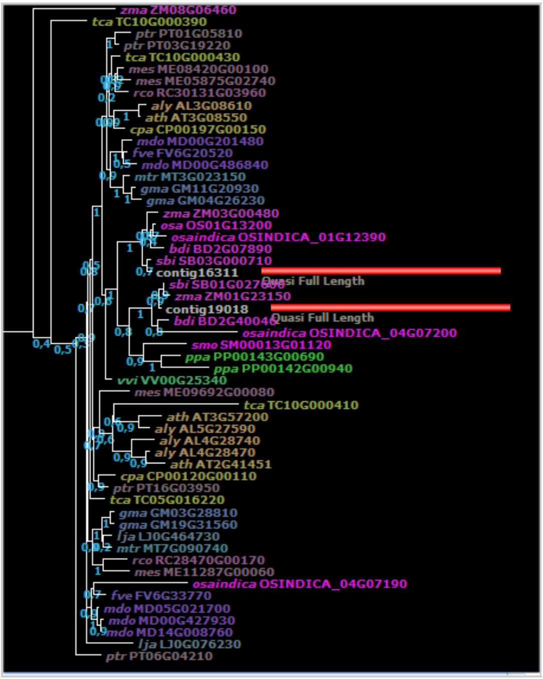

FIGURE 5 EXAMPLE FASTTREE PHYLOGENETIC TREE. (Panicum data set, transcript contig16311 in family HOM000957 covering 117 genes from 25 species,

more conserved alignment positions.

relaxed editing). The query transcript is shown in grey while homologs from the reference proteomes are shown in colors based on their taxonomic information. Meta-annotations are displayed as colored boxes next to the gene identifiers. Only a part of the complete tree is depicted in the image below.

In the TRAPID system we offer two different tree inference algorithms: FastTree (**http://meta.microbesonline.org/fasttree/**) and PhyMl (**http://www.atgc-montpellier.fr/phyml/**), with the first one being the default due to its very fast processing speed, coupled with equal or better fidelity (Price et al., 2010 PLoS One Mar 10;5(3):e9490). The user here has the ability to choose which algorithm to use, and -if desired- how many bootstrap runs will be applied. For FastTree we used the following non-default settings: '--wag gamma', which indicate that the algorithm uses the WAG+CAT model, and rescales the branch lengths. For PhyML we used the following non-default settings: '-m WAG -f e -c 4 -a e', which indicate that the algorithm uses the WAG+CAT model, that empirical amino acid frequencies are used, that 4 relative substitution rate categories are used, and that the parameter for the gamma distribution shape is based on the maximum likelihood estimate.

Finally, if the user has defined subsets within his/her experiment, these subsets can also be displayed on the phylogenetic tree, making subsequent analyses much easier. By default, the meta-annotation is displayed as domains next to the phylogenetic tree (see example below).

The default way to create a phylogenetic tree is:

1. Login into the TRAPID platform, and select the desired experiment
2. Select the transcript, either through the search function, or through any link in the platform
3. On the transcript page, select the associated gene family
4. On the gene family page, select the *create phylogenetic tree* from the toolbox
5. Select the reference species from the reference database you want to include in the tree

Step-by-step instructions on how to **construct phylogenetic trees** can be found in the tutorial.

Orthology

A key challenge in comparative genomics is reliably grouping homologous genes (derived from a common ancestor) and orthologous genes (homologs separated by a speciation event) into gene families. Orthology is generally considered a good proxy to identify genes performing a similar function in different species. Consequently, orthologs are frequently used as a means to transfer functional information from well-studied model systems to non-model organisms, for which e.g. only RNA-Seq-based gene catalogs are available. In eukaryotes, the utilization of orthology is not trivial, due to a wealth of paralogs (homologous genes created through a duplication event) in almost all lineages. Ancient duplication events preceding speciation led to outparalogs, which are frequently considered as subtypes within large gene families. In contrast to these are inparalogs, genes that originated through duplication events occurring after a speciation event. Besides continuous duplication events (for instance, via tandem duplication), many paralogs are remnants of different whole genome duplications (WGDs), resulting in the establishment of one-to-many and many-to-many orthologs (or co-orthologs).

Within TRAPID, the phylogenetic trees provide the most detailed approach to identify orthology relationships. For a given transcript, inspecting the phylogenetic tree can reveal whether orthologs exist in related species and if this relationship is a one-to-one, one-to-many or many-to-many orthology. Apart from the trees, also the *Browse similarity search output* tool, available from a transcript page, offers an overview of homologous genes in related species. Below, we show two examples demonstrating how orthologous groups and simple/complex orthology relationships can be derived from a phylogenetic tree generated using TRAPID.

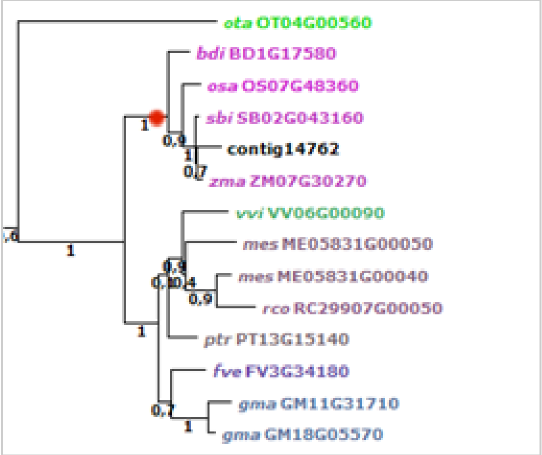

FIGURE 7 EXAMPLE ONE-TO-ONE ORTHOLOGY. (Panicum data set, transcript contig14762 RecQ helicase). The node indicated in red show s the monocot homologs for this clade (1 indicates 100% bootstrap support). Within this sub-tree, from each included monocot species a single gene is present, revealing simple one-to-one orthology relationships.

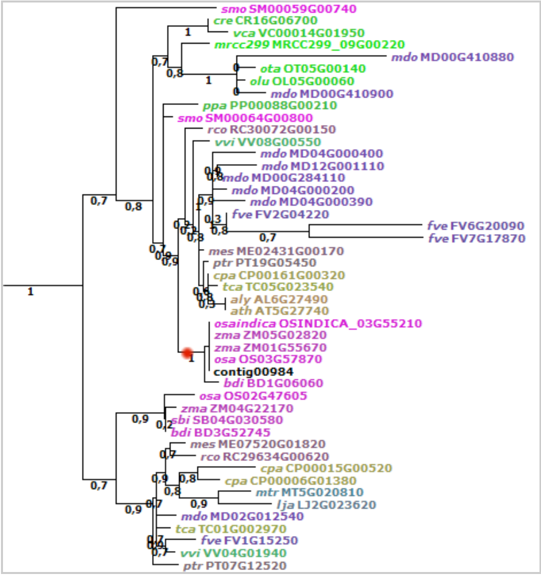

FIGURE 8 EXAMPLE ONE-TO-MANY ORTHOLOGY. (Panicum data set, transcript contig00984 ATPase). The node indicated in red show s the monocot homologs for this clade (1 indicates 100% bootstrap support). Within this sub-tree, 2 genes from *Zea mays* are present, revealing that for the single Panicum transcript tw o co-orthologs exist in *Z. mays*.

Remarks, suggestions or questions? Please contact the **Project leader**
