# Supplementary material for: TRAPID: an efficient online tool for the functional and comparative analysis of de novo RNA-Seq transcriptomes
Source: Genome Biol. 2013 Dec 13;14(12):R134. doi: 10.1186/gb-2013-14-12-r134 (PMC4053847; doi:10.1186/gb-2013-14-12-r134)
Supplement: Additional file 9 — TRAPID tutorial. [file gb-2013-14-12-r134-S9.pdf]

TRAPID: Rapid Analysis of Transcriptome Data

Documentation

TRAPID Tutorial

INTRODUCTION

TRAPID is an online tool for the fast and efficient processing of assembled RNA-Seq transcriptome data. TRAPID offers high-throughput ORF detection, frameshift correction and includes a functional, comparative and phylogenetic toolbox. Sequences can be ESTs, full-length cDNAs or RNA-Seq transcriptome sequences. Additionally, coding sequence derived from an annotated genome can also be used. We offer two reference databases: for plants and green algae **PLAZA 2.5**, for Alveolata, Amoebozoa, Euglenozoa, Fungi, Metazoa and prokaryotes (Bacteria and Archaea) **OrthoMCL-DB version 5** is available.

Once the initial processing has assigned functional annotations and gene families to the user-defined transcripts, evolutionary studies on gene families including the uploaded transcripts can be performed. Through a few simple operations, multiple sequence alignments and phylogenetic trees can be generated.

Although TRAPID hosts several prokaryotic reference genomes, it was not developed to process data from large-scale metagenomic studies.

SETTING UP AN ACCOUNT

Before you can use TRAPID, you need to register for an account. For academics this is free of charge. First click on the *"Register"* link (or click **here**) on the bottom of the page, on the next page fill in the required information and click on the *"Register"* button. Make sure to provide a valid e-mail address as your login credential will be send immediately by e-mail.

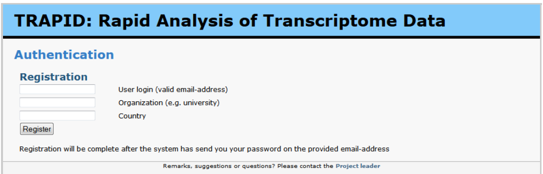

FIGURE 1 REGISTRATION FORM. Fill in your information and click register. An e-mail with login credentials will be send.

LOGIN TO YOUR ACCOUNT

On the main page, click *"Login"* (or click **here**) at the bottom of the page, this will take you to a login form. Here use the **e-mail address** used to register and the **password** sent by mail to login.

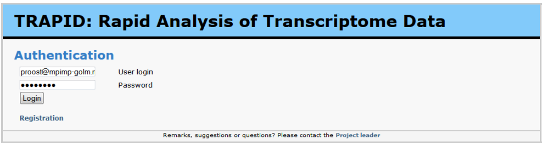

FIGURE 2 LOGIN FORM. Login using your e-mail address and the provided password.

TUTORIAL 1: FUNCTIONAL ANNOTATION OF PANICUM TRANSCRIPTS

PART 1: UPLOADING AND PROCESSING THE DATA

After login the main page will be replaced by the *Experiments overview*. If you are new this overview will be empty. Please note the sections *Current experiments* (experiments you uploaded and own), *Shared experiments* (experiments uploaded by others you are allowed to view) and *Add new experiment* where a new experiment can be started.

In this tutorial you'll learn how to functionally annotate the transcriptome of *Panicum hallii* (Meyer *et al*. 2012, Transcriptome analysis and gene expression atlas for *Panicum hallii* var. filipes, a diploid model for biofuel research.). The dataset can be obtained from : **ftp://ftp.psb.ugent.be/pub/trapid/**.

To start, like shown in Figure 3, enter *Tutorial 1* as a name and *Panicum transcripts* as description. Leave the Reference DB at its default setting *PLAZA 2.5*, this is the recommended database for plants and algae. In case data from other organisms is analyzed, select OrthoMCL-DB 5.0. Add the experiment by clicking *Create experiment*. The new experiment will now appear in the *Current experiments* list. Note that each user can have a **maximum of 10 experiments**.

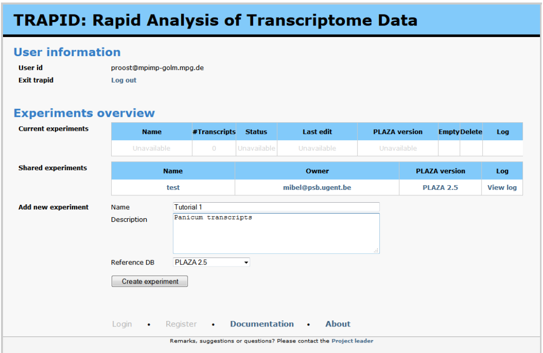

FIGURE 3 EXPERIMENT OVERVIEW. At the bottom a name and description for the new experiment needs to be filled in for the new experiment. Create the experiment by clicking *Create experiment*.

To continue, click on the experiment's name (*"Tutorial 1"*) in the *Current experiments* overview. This will take you to the *Experiment page*, here general statistics are shown (*Experiment information*), sequences can be imported and exported (*Import/Export*) and a search function is available to find specific sequences (*Search*). Further down are options to start the processing (*Initial processing*) and once data is added a toolbox will appear and a detailed overview of the transcripts.

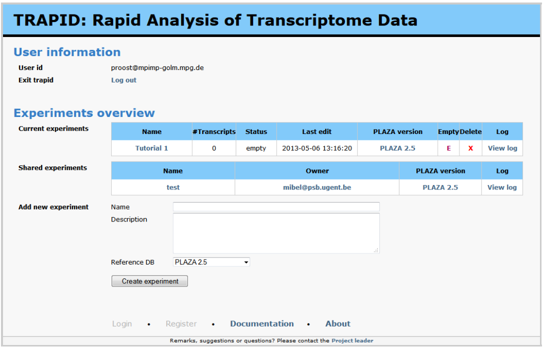

FIGURE 4 EXPERIMENT OVERVIEW. The new experiment appeared in the *"Current experiments"* list. To add sequences, first click on the name of the experiment.

Next click *Import data* to go to a page where the data can be uploaded (Transcript file management). All data needs to be provided as a fasta file. Make sure each sequence has a **unique identifier** (max. 50 characters) and no empty sequences are present. **Single files cannot be bigger than 32Mb**. For large datasets it is possible to offer the files as a (g)zipped file. Two options are available, in case you downloaded the tutorial dataset you can upload the file (use *Browse* to locate the file on your system), alternatively you can enter the URL **ftp://ftp.psb.ugent.be/pub/trapid/datasets/panicum/panicum_transcripts.zip**. To upload/get the dataset click *Import transcript sequences*. Once the data is available on our server, click *Load data from files into database* to get the sequences into our database. **Both steps are essential before the data can be processed.**

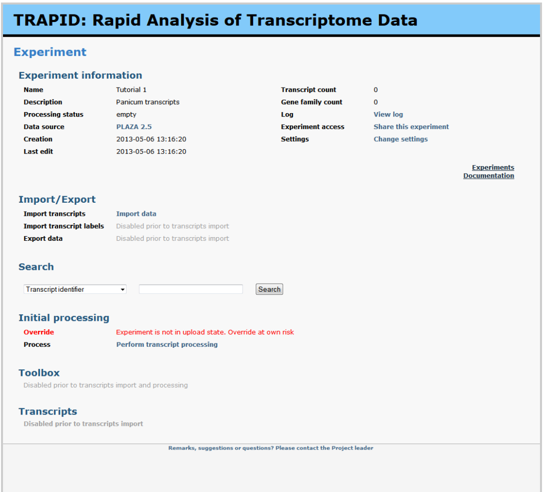

FIGURE 5 EXPERIMENT PAGE. The, currently still empty, experiment page. Click on *"Import data"* to import sequences.

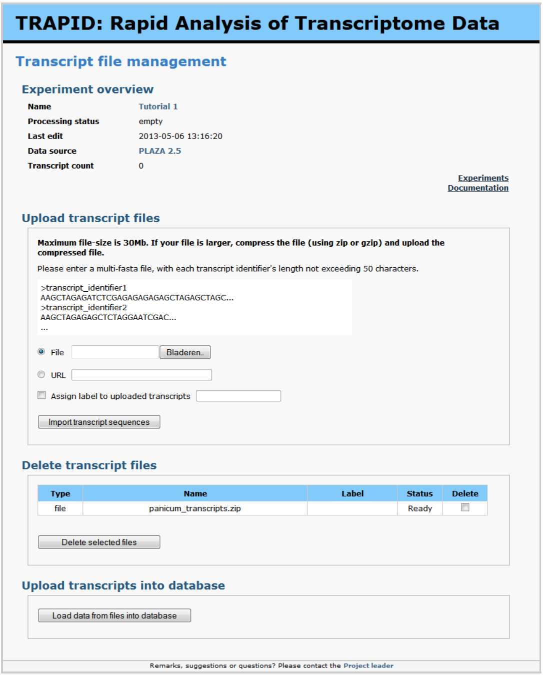

FIGURE 6 TRANSCRIPT FILE MANAGEMENT PAGE. From this page a dataset can be uploaded or directly grabbed from a URL. After the data is successfully transferred to our system please click *"Load data from files into database"*.

After the sequences have been loaded to the TRAPID database, the first thing that is required is to perform the initial processing. This step will add them to gene families and add functional annotation. Go to the experiment page (note how the *Toolbox* and *Transcripts* have become available, though they only become useful after the processing) and click *Perform transcript processing*. On the next page you have to specify how transcripts should be assigned to gene families. For this tutorial select the settings as shown in Figure 7. Finish by clicking *Start transcriptome pipeline*. Depending on the size of the dataset and the load on our servers this can take several hours. In the meanwhile the experiment will be in processing state and cannot be analyzed. **An e-mail will be send as soon as the dataset has been processed.**

Once the initial processing is done, all sequences will be included in gene families and will be functionally annotated (if possible). Additionally, as transcript data often included truncated sequences or sequences with indels, problematic sequences are flagged.

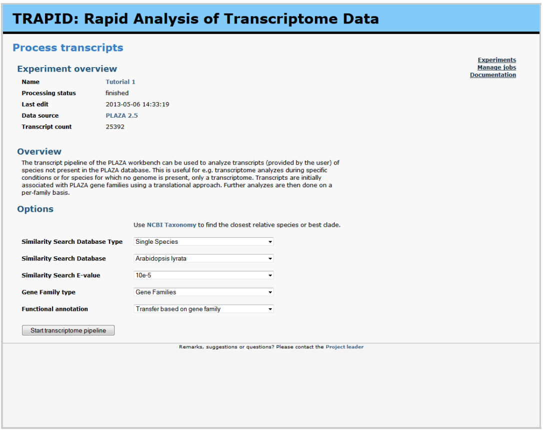

FIGURE 7 PROCESS TRANSCRIPTS. Select the desired settings how the sequences should be added to gene families and which funtional annotation should be transferred.

PART 2: EXPLORING TRAPID OUTPUT

Once the initial processing is completed, go back to the *"experiment overview"* page, options in the *"Toolbox"* are now available and at the bottom of the page a list of your sequences is shown with their gene family and predicted functional annotation. In the *Toolbox* click on *General statistics*. The following page will show a broad range of statistics that reveal the quality of the input dataset, how many sequences were

assigned to gene families and how many were functionally annotated (Figure 8). Other statistics available from the *Toolbox* are length distribution of the transcripts and the open reading frames.

Gene families (which group genes derived from a common ancestor) are available from the *Toolbox* under *Gene families*.

Relevant families can be found using the search function on the experiment page. E.g. by selecting *GO description* relevant GO labels can be found and the associated sequences can be found.

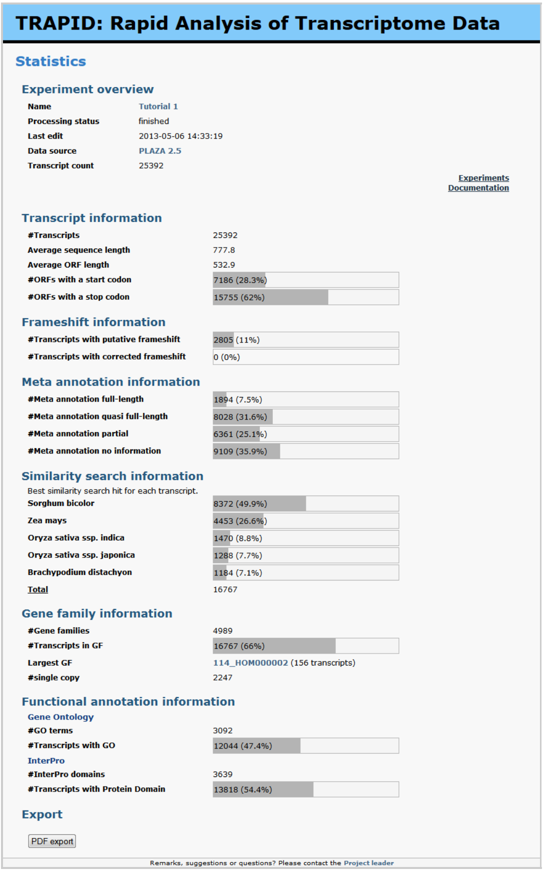

FIGURE 8 THE STATISTICS PAGE. Here general information is shown that can be used to assess the quality of the transcripts, how many were assigned to gene families and how many recieved funtional annotation. Also notice the *"pdf export"* button on the bottom of the page, this will generate a PDF with all shown information.

PART 3 PHYLOGENETIC ANALYSIS OF A SPECIFIC GENE FAMILY

First look for the GO term *leaf senescence* and look at the genes assigned this label in the dataset. Look at **contig04501**, this transcript is annotated as quasi full length. Now click the gene family ID next to the transcript identifier, this will take you to a gene family page. Build the multiple sequence alignment for this gene family by clicking *Create multiple sequence alignment* in the Toolbox. After the alignment is generated, a link will appear to start JalView to view the sequence alignment, or download the alignment. For this tutorial click *View full multiple sequence alignment* to start JalView. From this alignment can be seen the transcript has indeed a good alignment with most of the other members in the family, though at the N-terminal end there likely is a portion missing (and hence is indeed quasi full length). The other transcript (**contig20276**) is the opposite, here the C-terminal end appears to be missing, potentially both contigs represent a single, split transcript.

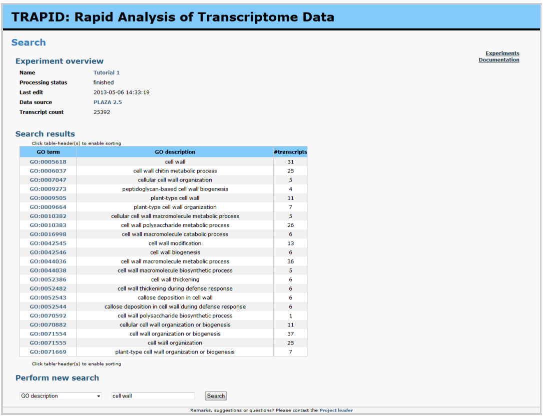

FIGURE 9 SEARCH RESULTS FOR TERM *"CELL WALL"* After searching on a GO description a list of corresponding go labels. from here quickly the associated sequences can be found.

Go back and find **contig01069**, find the gene family page of this transcript and in the toolbox select *Create phylogenetic tree*. On the next page, settings for the tree need to be set. Switch the Editing from *Stringent editing* to *Relaxed editing* (optionally fewer species can be selected). Next click *Create phylogenetic tree*. The job will be started and an e-mail will be send upon completion (usually within a few minutes). To view the tree, go back to the gene family page and click *Create phylogenetic tree* again. Now an ATV applet will be included on the page which shows the tree.

In case you don't see a tree or get an error message very often the number of

sequences is to large and should be lowered by excluding some species or the editing is too stringent and the relaxed editing should be selected. On the next page an ATV applet will show the phylogenetic tree. Please note that before generating a new tree with different settings the Java Cache needs to be cleared (for detailed instructions please check

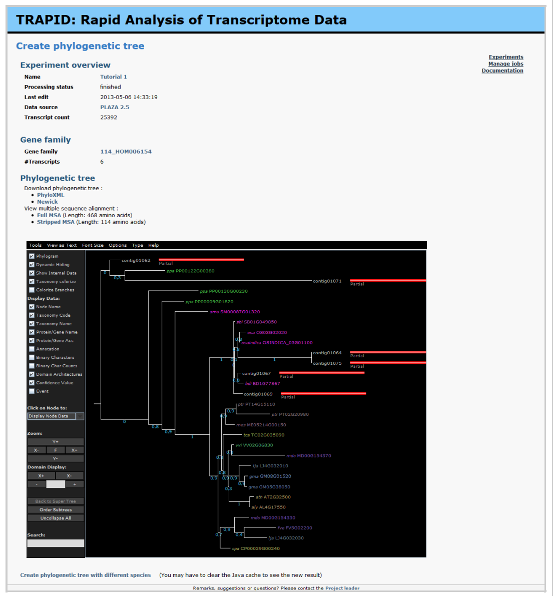

FIGURE 10 PHYLOGENETIC TREE ATV applet displaying the phylogenetic tree of contig01069 and its orthologs.

http://www.java.com/en/download/help/plugin_cache.xml).

From the phylogenetic tree page, the tree can be **downloaded in PhyloXML and Newick formats**.

Read more about **multiple sequence alignments** and **phylogenetic trees** in the general documentation

PART 4 ADD TRANSCRIPT LABELS AND ANALYZE EXPERIMENT SUBSETS (WITHIN-TRANSCRIPTOME FUNCTIONAL ANALYSIS)

If the data set is comprised of transcriptome data from different sources (with sources indicating different tissues, developmental types or stress conditions), then the user has the ability to assign labels to the subsets. This is done through the *import transcript labels* link on the experiment page. By doing so, several new analyses become available, such as comparison of functional annotation between different subsets, or by computing the enrichment factor compared to the overall transcriptome.

In the example here a set of Cell Cycle genes (list can be downloaded from

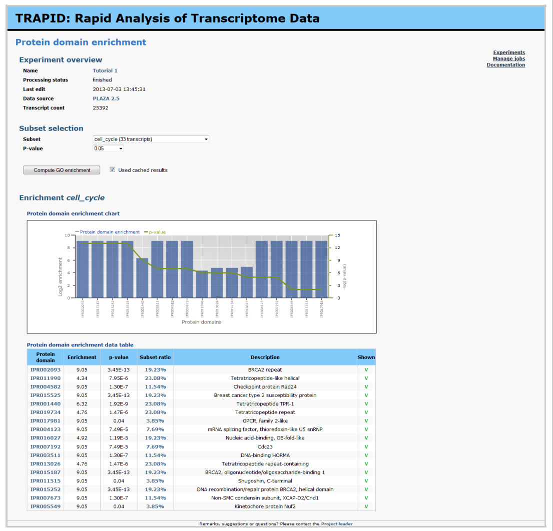

FIGURE 11 ENRICHED INTERPRO DOMAINS Overview of InterPro domain enrichted within the "Cell Cycle" subset

ftp://ftp.psb.ugent.be/pub/trapid/datasets/panicum/panicum_cell_cycle.lst). Add the labels to the dataset from the previous tutorial by clicking *Import data* next to *Import transcript labels*. On the next page, hit browse to select the file, enter a label (e.g. *Cell_cycle*) and click *Import labels*.

Go back to the experiment overview page, in the *Toolbox* a number of new options are enabled. To check if this set is enriched for specific GO terms or Protein domains, click *GO enrichment from a subset compared to background* or *Protein domain enrichment from a set compared to background*. For both GO and Protein domains, select the subset (here *Cell_cycle*) and the desired p-value and click *Compute enrichment*.

Figure 10 shows the resulting page with for each of the enriched InterPro domains the fold enrichment, significance and a short description. Note that the InterPro domain codes are hyperlinks to pages with more detailed information.

Read more about **labels** and **functional enrichment** in the general documentation

TUTORIAL 2: CORRECTING FRAMESHIFTS USING

FRAMEDP

In this second tutorial we'll use a single *Panicum* transcript which containing one indel (small insertion or deletion causing a frameshift, in this case a deletion), create a new experiment called *"Tutorial 2"* using the same data as tutorial 1. Next, start the transcript processing as shown in the previous tutorial using *"Eudicots"* as the phylogenetic clade. Once the processing is finished, go to the "Experiment overview" page and select a transcript (e.g. *contig17160*. The transcript page will show the line **"A putative frameshift was detected in this sequence"**. To attempt to correct this frameshift, select *"Correct framseshifts with FrameDP"* in the *"Toolbox"*. The next page will ask if you want to correct additional genes from the same family. This is not necessary here, so continue by clicking *"Perform frameshift correction"*. A new job is started and after a while you'll receive a notification via e-mail. After completion, TRAPID will indicate if a frameshift was corrected or not (Figure 12) and on the transcript page the corrected ORF can be obtained (Figure 13).

Read more about **frameshift correction** in the general documentation

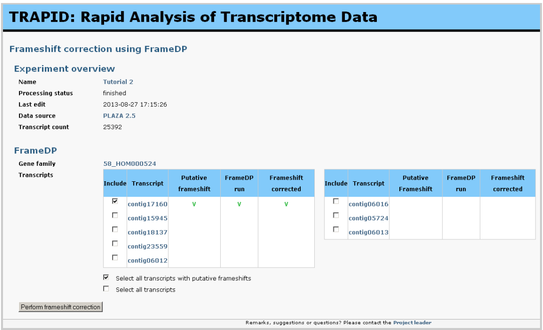

FIGURE 12 FRAMESHIFT CORRECTION. After FrameDP finishes the website will indicate a frameshift has succesfully been corrected.

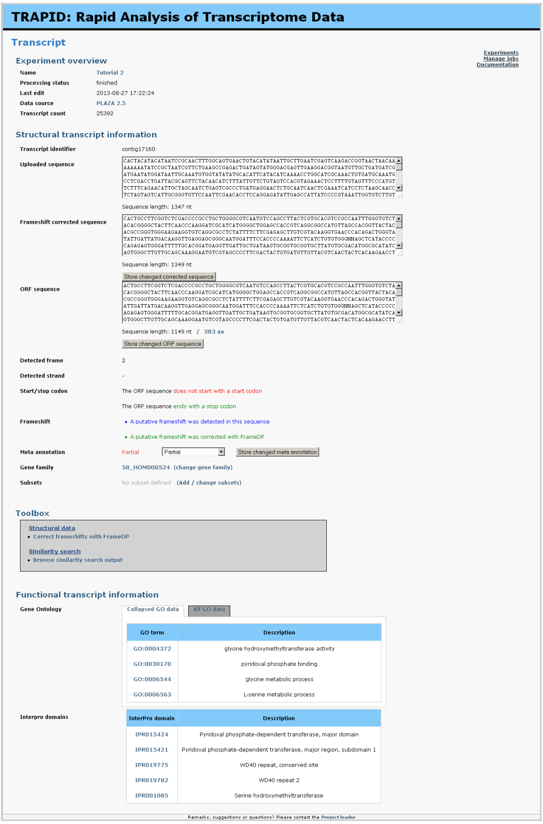

FIGURE 13 CORRECTED ORF SEQUENCE. In case a frameshift could be corrected the corrected sequence is available from the transcript page
